# The Al_61.49_Mn_11.35_Ni_4_ phase in the Al–Mn–Ni system

**DOI:** 10.1107/S2414314622000384

**Published:** 2022-01-14

**Authors:** Qifa Hu, Bin Wen, Changzeng Fan

**Affiliations:** a State Key Laboratory of Metastable Materials Science and Technology, Yanshan University, Qinhuangdao 066004, People’s Republic of China; Vienna University of Technology, Austria

**Keywords:** crystal structure, high-temperature sinter­ing, *R*-phase, Al–Mn–Ni system.

## Abstract

An inter­metallic phase in the Al–Mn–Ni system crystallizing in space group *Cmcm* (No. 63) and refined formula Al_61.49_Mn_11.35_Ni_4_ (called the *R*′ phase) has been synthesized by high-temperature sinter­ing of a mixture with initial chemical composition Al_60_Mn_7_Ni_3_. In comparison with the structure model of the previously reported *R* phase with composition Al_60_Mn_11_Ni_4_ [Robinson (1954 ▸). *Acta Cryst*. **7**, 494–497], there are two mutually exchanged Mn and Ni sites together with one positionally disordered Al site [occupancy ratio 0.811 (8):0.121 (7)] and one partially occupied Mn site [s.o.f. 0.677 (5)] in the current structure model of the *R*′ phase.

## Abstract 2

The Al_61.49_Mn_11.35_Ni_4_ phase was synthesized by high-temperature sinter­ing and its crystal structure has been re-determined.

## Graphical abstract

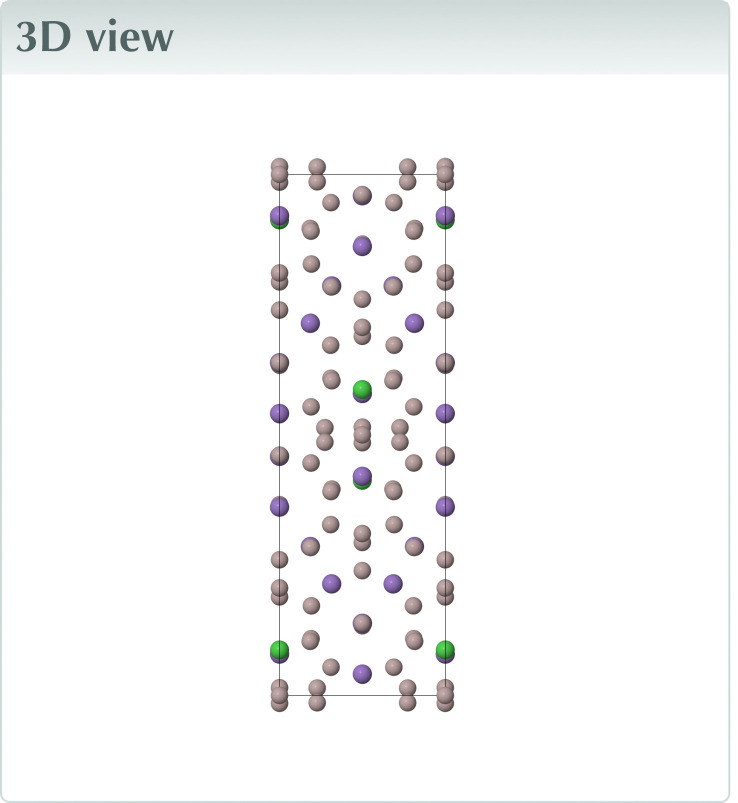

## Structure description

The ternary Al–Mn–Ni alloy system contains a variety of phases with complex or even quasicrystalline structures, most of which are not completely determined. Phase equilibria in the Al-rich region of the Al–Mn–Ni alloy system have been investigated previously. In this regard, a ternary phase with composition close to Al_60_Mn_11_Ni_4_ was reported as thermodynamically stable, crystallizing in space group *Bbmm* (non-conventional setting of space group *Cmcm*) with unit-cell parameters of *a* = 23.8, *b* = 12.5, *c* = 7.55 Å (Raynor, 1944 ▸). Its chemical composition was determined to be Al_80.0_Mn_14.7_Ni_5.3_ for the same sample. This phase was later denominated the *R* phase (Robinson, 1954 ▸). The derived crystal-structure model for the *R* phase had some ambiguities because at that time it was not possible to accurately model the deficiencies or the type of element for some of the atomic sites (Robinson, 1954 ▸). The *R* phase with similar composition/crystal structure has also been discovered in other systems, such as the *T*_3_ phase in the Al–Mn–Zn system or the Al_20_Mn_3_Cu_2_ phase (Damjanovic, 1961 ▸). It is inter­esting to note that the ortho­rhom­bic phase in the Al–Mn system is isostructural with the *R* phase and in coexistence with the deca­gonal quasicrystal in a rapidly solidified Al–Mn alloy, implying it is inseparable from the formation of quasicrystals (Li & Kuo, 1992 ▸).

In the present study, a slightly different crystal-structure model for the *R* phase in the Al–Mn–Ni system has been refined on basis of single-crystal X-ray diffraction data. This phase has similar unit-cell parameters to the previously reported *R* phase (Table 1 ▸, using the conventional setting *Cmcm*). Its chemical composition was refined to be Al_61.49_Mn_11.35_Ni_4_, in accordance with complementary SEM/EDX results (see Fig. S1 and Table S1 of the supporting information).

**Figure 1:**
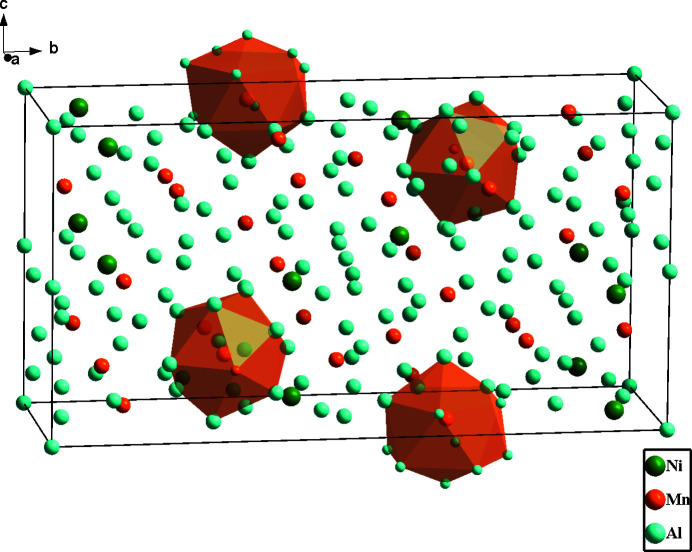
The crystal structure of Al_61.49_Mn_11.35_Ni_4_ with two Mn3 atoms and two Mn4 atoms displayed with their coordination environments as polyhedra.

**Figure 2:**
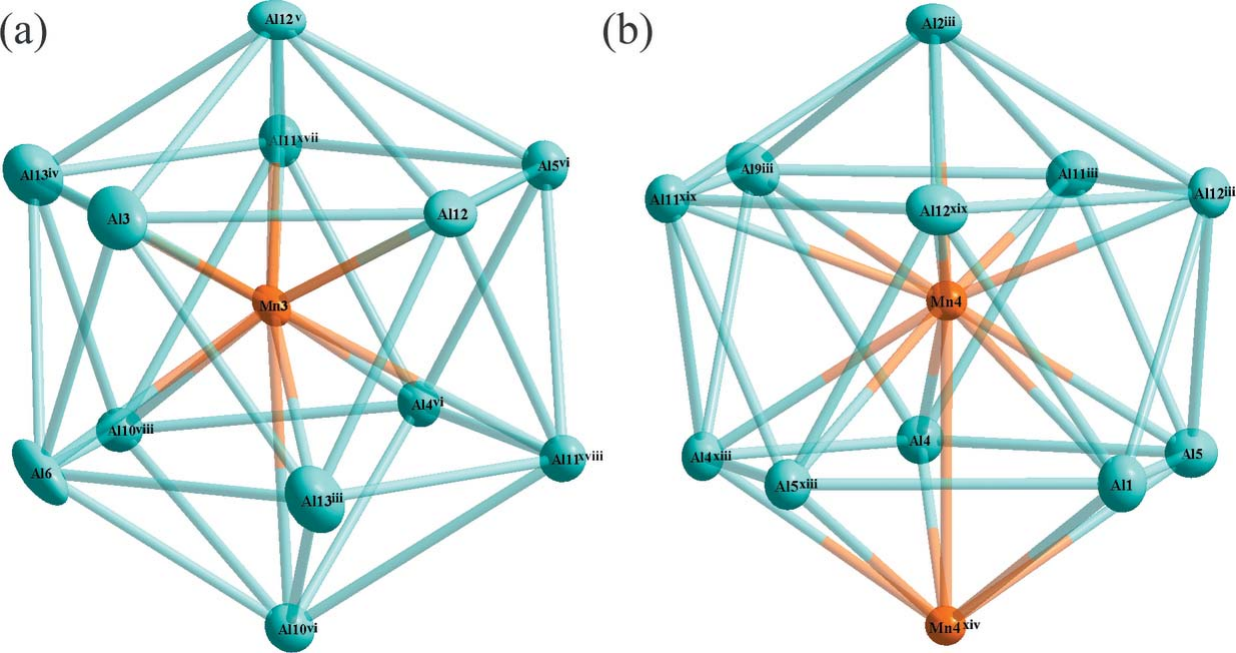
(*a*) The environment of the Mn3 atom at the 8*f* site; (*b*) the environment of the Mn4 atom at the 8*g* site with displacement ellipsoids given at the 90% probability level. [Symmetry codes: (iii) −x + 1/2, −*y* + 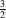 , −*z* + 1; (iv) *x* − 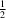 , −*y* + 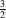 , −*z* + 1; (v) −*x*, *y*, *z*; (vi) *x*, *y*, *z* − 1; (viii) −*x*, *y*, *z* − 1; (xiii) *x*, *y*, −*z* + 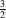 ; (xiv) −*x*, *y*, −*z* + 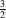 ; (xvii) *x* − 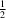 , −*y* + 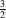 , −*z*; (xviii) −*x* + 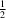 , −*y* + 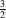 , −*z*; (xix) −*x* + 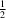 , −*y* + 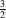 , *z* + 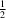 .]

**Figure 3:**
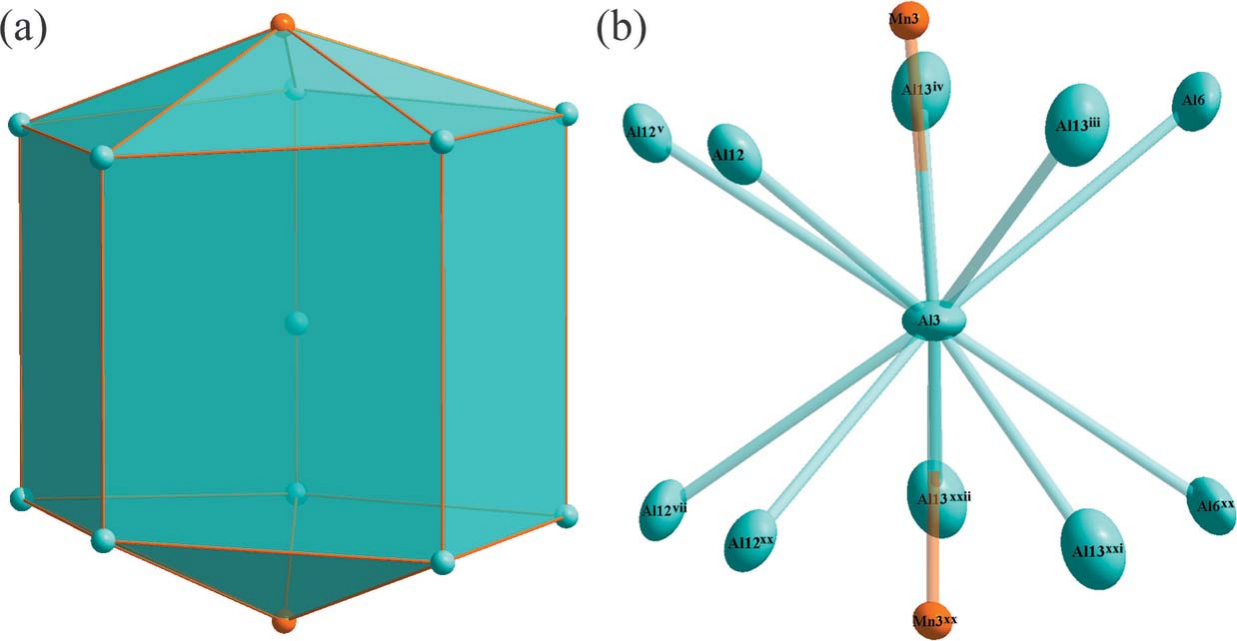
(*a*) The polyhedron formed around the Al3 atom at the 4*c* site; (*b*) the environment of the Al3 atom with displacement ellipsoids given at the 90% probability level. [Symmetry codes: (iii) −*x* + 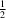 , −*y* + 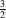 , −*z* + 1; (iv) *x* − 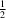 , −*y* + 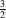 , −*z* + 1; (v) −*x*, *y*, *z*; (vii) −*x*, *y*, −*z* + 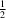 ; (xx) *x*, *y*, −*z* + 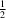 ; (xxi) −*x* + 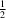 , −*y* + 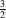 , *z* − 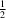 ; (xxii) *x* − 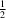 , −*y* + 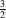 , *z* − 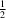 .]

In comparison with the *R* phase, the *R′* phase has a slightly higher Al and Mn content. A detailed comparison of the atomic labelling and coordinates between these two structure models along with the transformation matrix that transforms the original non-conventional setting to the current standard setting can be found in Table S2 of the supporting information. The *R*′ phase has two reversed sites compared to the original *R* phase whereby Mn4 in the original model becomes Ni1 in the current model, and *vice versa*. In addition, the *R*′ phase shows positional disorder of one Al site (Al7), and one Mn site (Mn2) with partial occupancy. Fig. 1 ▸ shows the distribution of all atoms in the unit cell of Al_61.49_Mn_11.35_Ni_4_ with four distorted icosa­hedra illustrated for simplicity. The environments of the Mn3 and Mn4 sites are shown in Fig. 2 ▸ *a* and 2*b*, respectively. The icosa­hedron centered at Mn3 is surrounded solely by Al atoms (Al3, Al4, Al5, Al6, Al10, Al11, Al12 and Al13) while that centered at Mn4 atom is composed by eleven Al atoms (Al1, Al2, Al4, Al5, Al9, Al11 and Al12) and one Mn atom (Mn4); all of the corresponding atomic sites are fully occupied. The polyhedron centered at Al3 is composed of a penta­gonal prism capped by two atoms at the base faces, as shown in Fig. 3 ▸ *a*. The environments of Al3 are displayed in Fig. 3 ▸ *b*, where ten Al atoms (Al6, Al12 and Al13) and two Mn atoms (Mn3) surround the central atom.

## Synthesis and crystallization

The high-purity elements Al (indicated purity 99.8%; 2.4285 g), Mn (indicated purity 99.96%; 0.5768 g) and Ni (indicated purity 99.9%; 0.2641 g) were mixed in the molar ratio 60:7:3 and ground in an agate mortar. The blended powders were placed into a cemented carbide grinding mound of 9.6 mm diameter and pressed at 4 MPa for about 5 min. The obtained cylindrical block was put into a silica glass tube and vacuum-sealed by a home-made sealing machine. The resulting ampoule then was placed in a furnace (SG-XQL1200) and heated up to 473 K for 10 min with a heating rate of 10 K min^−1^ and then heated up to 1373 K for 30 min with the same heating rate. Finally, the sample was slowly cooled to room temperature by turning off the furnace power. Suitable pieces of single-crystal grains were broken and selected from the product for single-crystal X-ray diffraction.

## Refinement

Crystal data, data collection and structure refinement details are summarized in Table 1 ▸. Manganese site Mn2 is partially occupied, and its site occupation factor (s.o.f.) was refined to 0.677 (5). The aluminium site Al17 was found to be disordered over two positions with refined s.o.f.s of 0.811 (8) and 0.121 (7) for Al7*A* and Al7*B*, respectively. The same anisotropic displacement parameters were used for these two split Al sites. All Ni sites in the present model show full occupancy. The maximum and minimum residual electron densities in the final difference map are located 1.42 Å from site Al11 and 0.57 Å from site Al7*A*, respectively.

## Supplementary Material

Crystal structure: contains datablock(s) I. DOI: 10.1107/S2414314622000384/wm4158sup1.cif

Structure factors: contains datablock(s) I. DOI: 10.1107/S2414314622000384/wm4158Isup2.hkl

supplementary materials. DOI: 10.1107/S2414314622000384/wm4158sup3.docx

reply. DOI: 10.1107/S2414314622000384/wm4158sup4.docx

CCDC reference: 2141311

Additional supporting information:  crystallographic information; 3D view; checkCIF report

## Figures and Tables

**Table 1:** Experimental details

Crystal data	
Chemical formula	Al_61.49_Mn_11.35_Ni_4_
*M*_r_	2517.49
Crystal system, space group	Orthorhombic, *C* *m* *c* *m*
Temperature (K)	296
*a*, *b*, *c* (Å)	7.6135 (3), 23.9582 (11), 12.4828 (6)
*V* (Å^3^)	2276.93 (18)
*Z*	2
Radiation type	Mo *K*α
μ (mm^−1^)	5.85
Crystal size (mm)	0.10 × 0.10 × 0.05

Data collection	
Diffractometer	Bruker D8 Venture Photon 100 CMOS
Absorption correction	Multi-scan (*SADABS*; Krause *et al.*, 2015 ▸)
*T*_min_, *T*_max_	0.648, 0.746
No. of measured, independent and observed [*I* > 2σ(*I*)] reflections	40442, 1584, 1269
*R*_int_	0.090
(sin θ/λ)_max_ (Å^−1^)	0.666

Refinement	
*R*[*F* ^2^ > 2σ(*F* ^2^)], *wR*(*F* ^2^), *S*	0.039, 0.095, 1.06
No. of reflections	1584
No. of parameters	114
Δρ_max_, Δρ_min_ (e Å^−3^)	1.85, −1.03

Computer programs: *APEX3* and *SAINT* (Bruker, 2015 ▸), *SHELXT* (Sheldrick, 2015*a* ▸), *SHELXL* (Sheldrick, 2015*b* ▸), *DIAMOND* (Brandenburg & Putz, 2017 ▸) and *publCIF* (Westrip, 2010 ▸).

## full crystallographic data

### Crystal data

**Table:**

Al_61.49_Mn_11.35_Ni_4_	*D*_x_ = 3.672 Mg m^−^^3^
*M**_r_* = 2517.49	Mo *K*α radiation, λ = 0.71073 Å
Orthorhombic, *C**m**c**m*	Cell parameters from 8707 reflections
*a* = 7.6135 (3) Å	θ = 2.4–30.6°
*b* = 23.9582 (11) Å	µ = 5.85 mm^−^^1^
*c* = 12.4828 (6) Å	*T* = 296 K
*V* = 2276.93 (18) Å^3^	Fragment, metallic
*Z* = 2	0.10 × 0.10 × 0.05 mm
*F*(000) = 2390	

### Data collection

**Table:**

Bruker D8 Venture Photon 100 CMOS diffractometer	1269 reflections with *I* > 2σ(*I*)
φ and ω scans	*R*_int_ = 0.090
Absorption correction: multi-scan (SADABS; Krause *et al.*, 2015)	θ_max_ = 28.3°, θ_min_ = 2.4°
*T*_min_ = 0.648, *T*_max_ = 0.746	*h* = −10→10
40442 measured reflections	*k* = −31→31
1584 independent reflections	*l* = −16→16

### Refinement

**Table:**

Refinement on *F*^2^	114 parameters
Least-squares matrix: full	0 restraints
*R*[*F*^2^ > 2σ(*F*^2^)] = 0.039	*w* = 1/[σ^2^(*F*_o_^2^) + (0.0463*P*)^2^ + 17.4467*P*] where *P* = (*F*_o_^2^ + 2*F*_c_^2^)/3
*wR*(*F*^2^) = 0.095	(Δ/σ)_max_ = 0.001
*S* = 1.06	Δρ_max_ = 1.85 e Å^−^^3^
1584 reflections	Δρ_min_ = −1.03 e Å^−^^3^

### Special details

**Table:**

Geometry. All esds (except the esd in the dihedral angle between two l.s. planes) are estimated using the full covariance matrix. The cell esds are taken into account individually in the estimation of esds in distances, angles and torsion angles; correlations between esds in cell parameters are only used when they are defined by crystal symmetry. An approximate (isotropic) treatment of cell esds is used for estimating esds involving l.s. planes.

### Fractional atomic coordinates and isotropic or equivalent isotropic displacement parameters (Å^2^)

**Table:**

	*x*	*y*	*z*	*U*_iso_*/*U*_eq_	Occ. (<1)
Ni1	0.000000	0.91273 (3)	0.06707 (7)	0.0123 (2)	
Mn1	0.000000	0.54113 (5)	0.750000	0.0070 (3)	
Mn2	0.000000	0.92062 (7)	0.750000	0.0071 (6)	0.677 (5)
Mn3	0.000000	0.63847 (3)	0.05426 (6)	0.00525 (19)	
Mn4	0.18574 (11)	0.71395 (3)	0.750000	0.00517 (19)	
Al1	0.000000	0.81074 (10)	0.750000	0.0081 (5)	
Al2	0.000000	0.79366 (10)	0.250000	0.0083 (5)	
Al3	0.000000	0.63386 (10)	0.250000	0.0111 (5)	
Al4	0.000000	0.63837 (6)	0.84947 (13)	0.0066 (3)	
Al5	0.000000	0.73945 (7)	0.93387 (13)	0.0076 (3)	
Al6	0.000000	0.53869 (7)	0.11718 (14)	0.0129 (4)	
Al7A	0.000000	0.98517 (9)	0.9064 (2)	0.0170 (8)	0.811 (8)
Al7B	0.000000	0.000000	0.000000	0.0170 (8)	0.121 (7)
Al8	0.2272 (3)	0.01406 (8)	0.250000	0.0250 (5)	
Al9	0.1846 (2)	0.89615 (7)	0.250000	0.0094 (4)	
Al10	0.18981 (16)	0.55406 (5)	0.93399 (9)	0.0075 (3)	
Al11	0.19159 (16)	0.82804 (5)	0.06539 (9)	0.0074 (3)	
Al12	0.18747 (16)	0.71601 (5)	0.12610 (9)	0.0080 (3)	
Al13	0.18943 (17)	0.89124 (5)	0.88587 (11)	0.0135 (3)	

### Atomic displacement parameters (Å^2^)

**Table:**

	*U*^11^	*U*^22^	*U*^33^	*U*^12^	*U*^13^	*U*^23^
Ni1	0.0055 (4)	0.0074 (4)	0.0241 (5)	0.000	0.000	−0.0016 (3)
Mn1	0.0134 (6)	0.0033 (5)	0.0044 (5)	0.000	0.000	0.000
Mn2	0.0057 (10)	0.0112 (10)	0.0043 (9)	0.000	0.000	0.000
Mn3	0.0080 (4)	0.0039 (4)	0.0039 (4)	0.000	0.000	0.0003 (3)
Mn4	0.0049 (4)	0.0059 (4)	0.0046 (4)	0.0000 (3)	0.000	0.000
Al1	0.0099 (12)	0.0074 (11)	0.0071 (11)	0.000	0.000	0.000
Al2	0.0051 (12)	0.0117 (12)	0.0080 (11)	0.000	0.000	0.000
Al3	0.0182 (14)	0.0106 (12)	0.0045 (11)	0.000	0.000	0.000
Al4	0.0074 (8)	0.0060 (8)	0.0065 (8)	0.000	0.000	0.0008 (6)
Al5	0.0077 (8)	0.0072 (8)	0.0079 (8)	0.000	0.000	0.0011 (6)
Al6	0.0241 (11)	0.0051 (8)	0.0094 (8)	0.000	0.000	0.0021 (6)
Al7A	0.0102 (12)	0.0087 (12)	0.0320 (15)	0.000	0.000	−0.0034 (10)
Al7B	0.0102 (12)	0.0087 (12)	0.0320 (15)	0.000	0.000	−0.0034 (10)
Al8	0.0427 (14)	0.0160 (10)	0.0162 (10)	0.0111 (9)	0.000	0.000
Al9	0.0088 (9)	0.0125 (8)	0.0068 (8)	−0.0031 (7)	0.000	0.000
Al10	0.0073 (6)	0.0069 (5)	0.0082 (6)	−0.0003 (4)	−0.0009 (5)	−0.0003 (4)
Al11	0.0073 (6)	0.0074 (5)	0.0074 (6)	0.0009 (4)	0.0006 (5)	0.0013 (4)
Al12	0.0077 (6)	0.0067 (5)	0.0098 (6)	−0.0012 (5)	0.0003 (5)	−0.0028 (4)
Al13	0.0130 (7)	0.0101 (6)	0.0174 (7)	−0.0008 (5)	−0.0003 (5)	0.0021 (5)

### Geometric parameters (Å, º)

**Table:**

Ni1—Al7B^i^	2.2523 (7)	Al2—Al9^vii^	2.829 (3)
Ni1—Al7A^ii^	2.468 (2)	Al2—Al9	2.829 (3)
Ni1—Al10^iii^	2.4921 (12)	Al2—Al11^xx^	2.8490 (13)
Ni1—Al10^iv^	2.4921 (12)	Al2—Al11^v^	2.8490 (13)
Ni1—Al11	2.4989 (13)	Al2—Al11^vii^	2.8490 (13)
Ni1—Al11^v^	2.4989 (13)	Al2—Al11	2.8490 (13)
Ni1—Al7A^vi^	2.652 (3)	Al3—Al6^xx^	2.819 (3)
Ni1—Al9	2.7106 (12)	Al3—Al6	2.819 (3)
Ni1—Al9^vii^	2.7107 (12)	Al3—Al12^xx^	2.881 (2)
Ni1—Al13^viii^	2.7315 (15)	Al3—Al12^vii^	2.881 (2)
Ni1—Al13^vi^	2.7315 (15)	Al3—Al12^v^	2.881 (2)
Mn1—Al8^ix^	2.462 (2)	Al3—Al12	2.881 (2)
Mn1—Al8^x^	2.462 (2)	Al3—Al13^iv^	2.9714 (14)
Mn1—Al6^xi^	2.5310 (19)	Al3—Al13^xxi^	2.9714 (14)
Mn1—Al6^xii^	2.5310 (19)	Al3—Al13^xxii^	2.9714 (14)
Mn1—Al4	2.6399 (19)	Al3—Al13^iii^	2.9714 (14)
Mn1—Al4^xiii^	2.6400 (19)	Al4—Al4^xiii^	2.483 (3)
Mn1—Al10^xiv^	2.7312 (12)	Al4—Al5	2.641 (2)
Mn1—Al10^v^	2.7312 (12)	Al4—Al10	2.6985 (18)
Mn1—Al10^xiii^	2.7312 (12)	Al4—Al10^v^	2.6985 (18)
Mn1—Al10	2.7312 (12)	Al4—Al11^iii^	2.7001 (15)
Mn1—Al9^iii^	2.8328 (18)	Al4—Al11^iv^	2.7001 (14)
Mn1—Al9^xv^	2.8328 (18)	Al4—Al9^xv^	2.8270 (18)
Mn2—Al8^xvi^	2.333 (2)	Al4—Al9^iii^	2.8270 (18)
Mn2—Al8^xii^	2.333 (2)	Al5—Al12^iii^	2.7131 (15)
Mn2—Al13^xiv^	2.3349 (14)	Al5—Al12^iv^	2.7131 (15)
Mn2—Al13^xiii^	2.3349 (14)	Al5—Al12^xxiii^	2.8480 (18)
Mn2—Al13^v^	2.3349 (14)	Al5—Al12^xxiv^	2.8480 (18)
Mn2—Al13	2.3349 (14)	Al5—Al11^iv^	2.8510 (15)
Mn2—Al7A^xiii^	2.491 (3)	Al5—Al11^iii^	2.8510 (15)
Mn2—Al7A	2.491 (3)	Al6—Al8^xxv^	2.722 (2)
Mn2—Al1	2.633 (3)	Al6—Al8^xxvi^	2.722 (2)
Mn3—Al3	2.4459 (8)	Al6—Al10^xii^	2.7266 (19)
Mn3—Al12^v^	2.5085 (13)	Al6—Al10^xxvii^	2.7266 (19)
Mn3—Al12	2.5086 (13)	Al6—Al10^viii^	2.7300 (19)
Mn3—Al6	2.5162 (19)	Al6—Al10^vi^	2.7300 (19)
Mn3—Al4^vi^	2.5563 (18)	Al6—Al13^iii^	2.9000 (16)
Mn3—Al13^iv^	2.5800 (14)	Al6—Al13^iv^	2.9000 (16)
Mn3—Al13^iii^	2.5800 (14)	Al7A—Al7A^xxviii^	2.442 (5)
Mn3—Al5^vi^	2.8481 (18)	Al7A—Al8^xii^	2.608 (3)
Mn3—Al11^xvii^	2.8962 (13)	Al7A—Al8^xvi^	2.608 (3)
Mn3—Al11^xviii^	2.8962 (13)	Al7A—Al13^v^	2.685 (2)
Mn3—Al10^viii^	2.9039 (13)	Al7A—Al13	2.685 (2)
Mn3—Al10^vi^	2.9039 (13)	Al7A—Al10^xxix^	2.9016 (17)
Mn4—Al2^iii^	2.3996 (9)	Al7A—Al10^xxvi^	2.9016 (17)
Mn4—Al12^iii^	2.4779 (13)	Al7B—Al10^xxx^	2.8166 (12)
Mn4—Al12^xix^	2.4779 (13)	Al7B—Al10^xxxi^	2.8166 (12)
Mn4—Al4	2.6116 (15)	Al7B—Al10^xxxii^	2.8166 (12)
Mn4—Al4^xiii^	2.6116 (15)	Al7B—Al10^x^	2.8166 (12)
Mn4—Al11^xix^	2.6823 (12)	Al8—Al9^xxxiii^	2.843 (3)
Mn4—Al11^iii^	2.6823 (12)	Al8—Al13^xxxiv^	2.847 (2)
Mn4—Al1	2.716 (2)	Al8—Al13^xxvii^	2.847 (2)
Mn4—Al5	2.7642 (15)	Al8—Al10^x^	2.8874 (16)
Mn4—Al5^xiii^	2.7642 (15)	Al8—Al10^xxxv^	2.8874 (16)
Mn4—Al9^iii^	2.8163 (19)	Al9—Al10^xxi^	2.7591 (15)
Mn4—Mn4^xiv^	2.8282 (17)	Al9—Al10^iii^	2.7591 (15)
Al1—Al5	2.861 (2)	Al9—Al9^vii^	2.811 (4)
Al1—Al5^xiii^	2.861 (2)	Al9—Al11	2.8242 (15)
Al1—Al12^xv^	2.9093 (14)	Al9—Al11^xx^	2.8242 (15)
Al1—Al12^iii^	2.9093 (14)	Al10—Al13^xxxvi^	2.7603 (17)
Al1—Al12^iv^	2.9093 (14)	Al10—Al10^v^	2.890 (2)
Al1—Al12^xix^	2.9093 (14)	Al11—Al13^vi^	2.7046 (17)
Al1—Al13	2.946 (2)	Al11—Al12^xviii^	2.7705 (16)
Al1—Al13^v^	2.946 (2)	Al11—Al12	2.7891 (16)
Al1—Al13^xiii^	2.946 (2)	Al11—Al11^v^	2.917 (2)
Al2—Al12	2.809 (2)	Al12—Al13^iii^	2.7393 (17)
Al2—Al12^v^	2.809 (2)	Al12—Al12^v^	2.855 (3)
Al2—Al12^vii^	2.809 (2)	Al13—Al13^v^	2.884 (3)
Al2—Al12^xx^	2.809 (2)		
			
Al7A^ii^—Ni1—Al10^iii^	71.59 (3)	Al11^iv^—Al4—Al9^iii^	177.12 (7)
Al7A^ii^—Ni1—Al10^iv^	71.59 (3)	Al9^xv^—Al4—Al9^iii^	116.30 (7)
Al10^iii^—Ni1—Al10^iv^	142.75 (6)	Al4—Al5—Al12^iii^	104.51 (5)
Al7B^i^—Ni1—Al11	138.65 (3)	Al4—Al5—Al12^iv^	104.51 (5)
Al7A^ii^—Ni1—Al11	143.68 (3)	Al12^iii^—Al5—Al12^iv^	122.57 (8)
Al10^iii^—Ni1—Al11	72.91 (4)	Al4—Al5—Mn4	57.73 (5)
Al10^iv^—Ni1—Al11	144.33 (5)	Al12^iii^—Al5—Mn4	53.78 (4)
Al7A^ii^—Ni1—Al11^v^	143.68 (3)	Al12^iv^—Al5—Mn4	107.85 (6)
Al10^iii^—Ni1—Al11^v^	144.33 (5)	Al4—Al5—Mn4^xiv^	57.73 (5)
Al10^iv^—Ni1—Al11^v^	72.91 (4)	Al12^iii^—Al5—Mn4^xiv^	107.85 (6)
Al11—Ni1—Al11^v^	71.42 (6)	Al12^iv^—Al5—Mn4^xiv^	53.78 (4)
Al7A^ii^—Ni1—Al7A^vi^	56.83 (10)	Mn4—Al5—Mn4^xiv^	61.54 (5)
Al10^iii^—Ni1—Al7A^vi^	77.70 (3)	Al4—Al5—Al12^xxiii^	98.93 (6)
Al10^iv^—Ni1—Al7A^vi^	77.70 (3)	Al12^iii^—Al5—Al12^xxiii^	82.56 (4)
Al11—Ni1—Al7A^vi^	121.67 (5)	Al12^iv^—Al5—Al12^xxiii^	138.55 (6)
Al11^v^—Ni1—Al7A^vi^	121.67 (5)	Mn4—Al5—Al12^xxiii^	113.56 (4)
Al7B^i^—Ni1—Al9	116.69 (4)	Mn4^xiv^—Al5—Al12^xxiii^	155.84 (7)
Al7A^ii^—Ni1—Al9	91.85 (7)	Al4—Al5—Al12^xxiv^	98.93 (6)
Al10^iii^—Ni1—Al9	63.89 (4)	Al12^iii^—Al5—Al12^xxiv^	138.55 (6)
Al10^iv^—Ni1—Al9	122.86 (5)	Al12^iv^—Al5—Al12^xxiv^	82.56 (4)
Al11—Ni1—Al9	65.51 (4)	Mn4—Al5—Al12^xxiv^	155.84 (7)
Al11^v^—Ni1—Al9	101.00 (5)	Mn4^xiv^—Al5—Al12^xxiv^	113.56 (4)
Al7A^vi^—Ni1—Al9	137.13 (5)	Al12^xxiii^—Al5—Al12^xxiv^	60.15 (6)
Al7A^ii^—Ni1—Al9^vii^	91.85 (7)	Al4—Al5—Mn3^xxiii^	55.36 (5)
Al10^iii^—Ni1—Al9^vii^	122.86 (5)	Al12^iii^—Al5—Mn3^xxiii^	118.66 (4)
Al10^iv^—Ni1—Al9^vii^	63.89 (4)	Al12^iv^—Al5—Mn3^xxiii^	118.66 (4)
Al11—Ni1—Al9^vii^	100.99 (5)	Mn4—Al5—Mn3^xxiii^	104.50 (5)
Al11^v^—Ni1—Al9^vii^	65.51 (4)	Mn4^xiv^—Al5—Mn3^xxiii^	104.50 (5)
Al7A^vi^—Ni1—Al9^vii^	137.13 (5)	Al12^xxiii^—Al5—Mn3^xxiii^	52.26 (4)
Al9—Ni1—Al9^vii^	62.46 (7)	Al12^xxiv^—Al5—Mn3^xxiii^	52.26 (4)
Al7A^ii^—Ni1—Al13^viii^	107.32 (6)	Al4—Al5—Al11^iv^	58.75 (4)
Al10^iii^—Ni1—Al13^viii^	123.79 (5)	Al12^iii^—Al5—Al11^iv^	161.14 (7)
Al10^iv^—Ni1—Al13^viii^	63.61 (4)	Al12^iv^—Al5—Al11^iv^	60.11 (4)
Al11—Ni1—Al13^viii^	98.53 (4)	Mn4—Al5—Al11^iv^	107.38 (6)
Al11^v^—Ni1—Al13^viii^	62.09 (4)	Mn4^xiv^—Al5—Al11^iv^	57.04 (3)
Al7A^vi^—Ni1—Al13^viii^	59.81 (5)	Al12^xxiii^—Al5—Al11^iv^	107.35 (6)
Al9—Ni1—Al13^viii^	160.69 (5)	Al12^xxiv^—Al5—Al11^iv^	58.17 (4)
Al9^vii^—Ni1—Al13^viii^	113.34 (4)	Mn3^xxiii^—Al5—Al11^iv^	61.09 (4)
Al7A^ii^—Ni1—Al13^vi^	107.32 (6)	Al4—Al5—Al11^iii^	58.75 (4)
Al10^iii^—Ni1—Al13^vi^	63.61 (4)	Al12^iii^—Al5—Al11^iii^	60.11 (4)
Al10^iv^—Ni1—Al13^vi^	123.79 (5)	Al12^iv^—Al5—Al11^iii^	161.14 (7)
Al11—Ni1—Al13^vi^	62.09 (4)	Mn4—Al5—Al11^iii^	57.04 (3)
Al11^v^—Ni1—Al13^vi^	98.53 (4)	Mn4^xiv^—Al5—Al11^iii^	107.38 (6)
Al7A^vi^—Ni1—Al13^vi^	59.81 (5)	Al12^xxiii^—Al5—Al11^iii^	58.17 (4)
Al9—Ni1—Al13^vi^	113.34 (4)	Al12^xxiv^—Al5—Al11^iii^	107.35 (6)
Al9^vii^—Ni1—Al13^vi^	160.69 (5)	Mn3^xxiii^—Al5—Al11^iii^	61.09 (4)
Al13^viii^—Ni1—Al13^vi^	63.74 (6)	Al11^iv^—Al5—Al11^iii^	110.89 (7)
Al8^ix^—Mn1—Al8^x^	115.05 (12)	Al4—Al5—Al1	103.14 (8)
Al8^ix^—Mn1—Al6^xi^	66.07 (5)	Al12^iii^—Al5—Al1	62.86 (4)
Al8^x^—Mn1—Al6^xi^	66.07 (5)	Al12^iv^—Al5—Al1	62.86 (4)
Al8^ix^—Mn1—Al6^xii^	66.07 (5)	Mn4—Al5—Al1	57.71 (5)
Al8^x^—Mn1—Al6^xii^	66.07 (5)	Mn4^xiv^—Al5—Al1	57.71 (5)
Al6^xi^—Mn1—Al6^xii^	81.85 (9)	Al12^xxiii^—Al5—Al1	142.53 (5)
Al8^ix^—Mn1—Al4	118.28 (5)	Al12^xxiv^—Al5—Al1	142.53 (5)
Al8^x^—Mn1—Al4	118.28 (5)	Mn3^xxiii^—Al5—Al1	158.50 (8)
Al6^xi^—Mn1—Al4	167.13 (6)	Al11^iv^—Al5—Al1	109.95 (5)
Al6^xii^—Mn1—Al4	111.02 (5)	Al11^iii^—Al5—Al1	109.95 (5)
Al8^ix^—Mn1—Al4^xiii^	118.28 (5)	Mn3—Al6—Mn1^xii^	157.26 (8)
Al8^x^—Mn1—Al4^xiii^	118.28 (5)	Mn3—Al6—Al8^xxv^	113.33 (6)
Al6^xi^—Mn1—Al4^xiii^	111.02 (5)	Mn1^xii^—Al6—Al8^xxv^	55.75 (5)
Al6^xii^—Mn1—Al4^xiii^	167.13 (6)	Mn3—Al6—Al8^xxvi^	113.33 (6)
Al4—Mn1—Al4^xiii^	56.11 (7)	Mn1^xii^—Al6—Al8^xxvi^	55.75 (5)
Al8^ix^—Mn1—Al10^xiv^	67.33 (3)	Al8^xxv^—Al6—Al8^xxvi^	99.45 (9)
Al8^x^—Mn1—Al10^xiv^	120.48 (3)	Mn3—Al6—Al10^xii^	134.52 (6)
Al6^xi^—Mn1—Al10^xiv^	62.28 (4)	Mn1^xii^—Al6—Al10^xii^	62.46 (4)
Al6^xii^—Mn1—Al10^xiv^	129.53 (5)	Al8^xxv^—Al6—Al10^xii^	111.74 (7)
Al4—Mn1—Al10^xiv^	107.18 (5)	Al8^xxvi^—Al6—Al10^xii^	64.00 (5)
Al4^xiii^—Mn1—Al10^xiv^	60.29 (4)	Mn3—Al6—Al10^xxvii^	134.52 (6)
Al8^ix^—Mn1—Al10^v^	67.33 (3)	Mn1^xii^—Al6—Al10^xxvii^	62.46 (4)
Al8^x^—Mn1—Al10^v^	120.48 (3)	Al8^xxv^—Al6—Al10^xxvii^	64.00 (5)
Al6^xi^—Mn1—Al10^v^	129.53 (5)	Al8^xxvi^—Al6—Al10^xxvii^	111.74 (7)
Al6^xii^—Mn1—Al10^v^	62.28 (4)	Al10^xii^—Al6—Al10^xxvii^	64.01 (6)
Al4—Mn1—Al10^v^	60.29 (4)	Mn3—Al6—Al10^viii^	67.07 (5)
Al4^xiii^—Mn1—Al10^v^	107.18 (5)	Mn1^xii^—Al6—Al10^viii^	130.58 (6)
Al10^xiv^—Mn1—Al10^v^	114.48 (5)	Al8^xxv^—Al6—Al10^viii^	160.60 (7)
Al8^ix^—Mn1—Al10^xiii^	120.48 (3)	Al8^xxvi^—Al6—Al10^viii^	97.78 (4)
Al8^x^—Mn1—Al10^xiii^	67.33 (3)	Al10^xii^—Al6—Al10^viii^	68.47 (6)
Al6^xi^—Mn1—Al10^xiii^	62.28 (4)	Al10^xxvii^—Al6—Al10^viii^	101.20 (7)
Al6^xii^—Mn1—Al10^xiii^	129.53 (5)	Mn3—Al6—Al10^vi^	67.07 (5)
Al4—Mn1—Al10^xiii^	107.18 (5)	Mn1^xii^—Al6—Al10^vi^	130.58 (6)
Al4^xiii^—Mn1—Al10^xiii^	60.29 (4)	Al8^xxv^—Al6—Al10^vi^	97.78 (4)
Al10^xiv^—Mn1—Al10^xiii^	63.89 (5)	Al8^xxvi^—Al6—Al10^vi^	160.60 (7)
Al10^v^—Mn1—Al10^xiii^	166.98 (7)	Al10^xii^—Al6—Al10^vi^	101.20 (7)
Al8^ix^—Mn1—Al10	120.48 (3)	Al10^xxvii^—Al6—Al10^vi^	68.47 (6)
Al8^x^—Mn1—Al10	67.33 (3)	Al10^viii^—Al6—Al10^vi^	63.92 (6)
Al6^xi^—Mn1—Al10	129.54 (5)	Mn3—Al6—Al3	54.21 (5)
Al6^xii^—Mn1—Al10	62.28 (4)	Mn1^xii^—Al6—Al3	103.05 (7)
Al4—Mn1—Al10	60.29 (4)	Al8^xxv^—Al6—Al3	79.47 (6)
Al4^xiii^—Mn1—Al10	107.18 (5)	Al8^xxvi^—Al6—Al3	79.47 (6)
Al10^xiv^—Mn1—Al10	166.98 (7)	Al10^xii^—Al6—Al3	142.84 (5)
Al10^v^—Mn1—Al10	63.89 (5)	Al10^xxvii^—Al6—Al3	142.84 (5)
Al10^xiii^—Mn1—Al10	114.48 (5)	Al10^viii^—Al6—Al3	112.56 (6)
Al8^ix^—Mn1—Al9^iii^	179.56 (8)	Al10^vi^—Al6—Al3	112.56 (6)
Al8^x^—Mn1—Al9^iii^	64.51 (6)	Mn3—Al6—Al13^iii^	56.36 (4)
Al6^xi^—Mn1—Al9^iii^	113.63 (3)	Mn1^xii^—Al6—Al13^iii^	116.48 (5)
Al6^xii^—Mn1—Al9^iii^	113.63 (3)	Al8^xxv^—Al6—Al13^iii^	60.75 (5)
Al4—Mn1—Al9^iii^	62.08 (4)	Al8^xxvi^—Al6—Al13^iii^	139.08 (8)
Al4^xiii^—Mn1—Al9^iii^	62.09 (4)	Al10^xii^—Al6—Al13^iii^	154.26 (6)
Al10^xiv^—Mn1—Al9^iii^	112.85 (4)	Al10^xxvii^—Al6—Al13^iii^	92.09 (4)
Al10^v^—Mn1—Al9^iii^	112.85 (4)	Al10^viii^—Al6—Al13^iii^	110.04 (7)
Al10^xiii^—Mn1—Al9^iii^	59.42 (3)	Al10^vi^—Al6—Al13^iii^	58.63 (4)
Al10—Mn1—Al9^iii^	59.42 (3)	Al3—Al6—Al13^iii^	62.59 (4)
Al8^ix^—Mn1—Al9^xv^	64.51 (6)	Mn3—Al6—Al13^iv^	56.36 (4)
Al8^x^—Mn1—Al9^xv^	179.56 (8)	Mn1^xii^—Al6—Al13^iv^	116.48 (5)
Al6^xi^—Mn1—Al9^xv^	113.63 (3)	Al8^xxv^—Al6—Al13^iv^	139.08 (8)
Al6^xii^—Mn1—Al9^xv^	113.63 (3)	Al8^xxvi^—Al6—Al13^iv^	60.75 (5)
Al4—Mn1—Al9^xv^	62.09 (4)	Al10^xii^—Al6—Al13^iv^	92.09 (4)
Al4^xiii^—Mn1—Al9^xv^	62.09 (4)	Al10^xxvii^—Al6—Al13^iv^	154.26 (6)
Al10^xiv^—Mn1—Al9^xv^	59.42 (3)	Al10^viii^—Al6—Al13^iv^	58.63 (4)
Al10^v^—Mn1—Al9^xv^	59.42 (3)	Al10^vi^—Al6—Al13^iv^	110.04 (7)
Al10^xiii^—Mn1—Al9^xv^	112.85 (4)	Al3—Al6—Al13^iv^	62.59 (4)
Al10—Mn1—Al9^xv^	112.85 (4)	Al13^iii^—Al6—Al13^iv^	109.24 (7)
Al9^iii^—Mn1—Al9^xv^	115.92 (8)	Al7A^xxviii^—Al7A—Ni1^ii^	65.38 (9)
Al8^xvi^—Mn2—Al8^xii^	95.71 (13)	Al7A^xxviii^—Al7A—Mn2	158.53 (16)
Al8^xvi^—Mn2—Al13^xiv^	131.32 (5)	Ni1^ii^—Al7A—Mn2	136.09 (12)
Al8^xii^—Mn2—Al13^xiv^	75.18 (5)	Al7A^xxviii^—Al7A—Al8^xii^	135.57 (7)
Al8^xvi^—Mn2—Al13^xiii^	75.18 (5)	Ni1^ii^—Al7A—Al8^xii^	95.36 (8)
Al8^xii^—Mn2—Al13^xiii^	131.32 (5)	Mn2—Al7A—Al8^xii^	54.39 (7)
Al13^xiv^—Mn2—Al13^xiii^	76.30 (7)	Al7A^xxviii^—Al7A—Al8^xvi^	135.57 (7)
Al8^xvi^—Mn2—Al13^v^	131.32 (5)	Ni1^ii^—Al7A—Al8^xvi^	95.36 (8)
Al8^xii^—Mn2—Al13^v^	75.18 (5)	Mn2—Al7A—Al8^xvi^	54.39 (7)
Al13^xiv^—Mn2—Al13^v^	93.17 (7)	Al8^xii^—Al7A—Al8^xvi^	83.07 (11)
Al13^xiii^—Mn2—Al13^v^	144.92 (10)	Al7A^xxviii^—Al7A—Ni1^xxiii^	57.79 (10)
Al8^xvi^—Mn2—Al13	75.18 (5)	Ni1^ii^—Al7A—Ni1^xxiii^	123.17 (10)
Al8^xii^—Mn2—Al13	131.32 (5)	Mn2—Al7A—Ni1^xxiii^	100.74 (8)
Al13^xiv^—Mn2—Al13	144.91 (10)	Al8^xii^—Al7A—Ni1^xxiii^	124.80 (8)
Al13^xiii^—Mn2—Al13	93.16 (7)	Al8^xvi^—Al7A—Ni1^xxiii^	124.80 (8)
Al13^v^—Mn2—Al13	76.30 (7)	Al7A^xxviii^—Al7A—Al13^v^	109.59 (12)
Al8^xvi^—Mn2—Al7A^xiii^	65.38 (5)	Ni1^ii^—Al7A—Al13^v^	147.49 (4)
Al8^xii^—Mn2—Al7A^xiii^	65.38 (5)	Mn2—Al7A—Al13^v^	53.47 (6)
Al13^xiv^—Mn2—Al7A^xiii^	67.53 (5)	Al8^xii^—Al7A—Al13^v^	65.06 (7)
Al13^xiii^—Mn2—Al7A^xiii^	67.53 (5)	Al8^xvi^—Al7A—Al13^v^	106.89 (10)
Al13^v^—Mn2—Al7A^xiii^	139.16 (5)	Ni1^xxiii^—Al7A—Al13^v^	61.56 (6)
Al13—Mn2—Al7A^xiii^	139.16 (5)	Al7A^xxviii^—Al7A—Al13	109.59 (12)
Al8^xvi^—Mn2—Al7A	65.38 (6)	Ni1^ii^—Al7A—Al13	147.49 (4)
Al8^xii^—Mn2—Al7A	65.38 (5)	Mn2—Al7A—Al13	53.47 (6)
Al13^xiv^—Mn2—Al7A	139.16 (5)	Al8^xii^—Al7A—Al13	106.89 (10)
Al13^xiii^—Mn2—Al7A	139.16 (5)	Al8^xvi^—Al7A—Al13	65.06 (7)
Al13^v^—Mn2—Al7A	67.53 (5)	Ni1^xxiii^—Al7A—Al13	61.56 (6)
Al13—Mn2—Al7A	67.53 (5)	Al13^v^—Al7A—Al13	64.98 (7)
Al7A^xiii^—Mn2—Al7A	103.23 (13)	Al7A^xxviii^—Al7A—Al10^xxix^	73.80 (7)
Al8^xvi^—Mn2—Al1	132.14 (7)	Ni1^ii^—Al7A—Al10^xxix^	54.58 (4)
Al8^xii^—Mn2—Al1	132.14 (7)	Mn2—Al7A—Al10^xxix^	116.50 (6)
Al13^xiv^—Mn2—Al1	72.46 (5)	Al8^xii^—Al7A—Al10^xxix^	128.64 (11)
Al13^xiii^—Mn2—Al1	72.46 (5)	Al8^xvi^—Al7A—Al10^xxix^	62.94 (5)
Al13^v^—Mn2—Al1	72.46 (5)	Ni1^xxiii^—Al7A—Al10^xxix^	106.41 (7)
Al13—Mn2—Al1	72.46 (5)	Al13^v^—Al7A—Al10^xxix^	157.69 (7)
Al7A^xiii^—Mn2—Al1	128.38 (6)	Al13—Al7A—Al10^xxix^	92.91 (4)
Al7A—Mn2—Al1	128.38 (6)	Al7A^xxviii^—Al7A—Al10^xxvi^	73.80 (7)
Al3—Mn3—Al12^v^	71.11 (6)	Ni1^ii^—Al7A—Al10^xxvi^	54.58 (4)
Al3—Mn3—Al12	71.11 (6)	Mn2—Al7A—Al10^xxvi^	116.50 (6)
Al12^v^—Mn3—Al12	69.36 (6)	Al8^xii^—Al7A—Al10^xxvi^	62.94 (5)
Al3—Mn3—Al6	69.22 (7)	Al8^xvi^—Al7A—Al10^xxvi^	128.64 (11)
Al12^v^—Mn3—Al6	126.30 (5)	Ni1^xxiii^—Al7A—Al10^xxvi^	106.41 (7)
Al12—Mn3—Al6	126.30 (5)	Al13^v^—Al7A—Al10^xxvi^	92.91 (4)
Al3—Mn3—Al4^vi^	177.36 (8)	Al13—Al7A—Al10^xxvi^	157.69 (7)
Al12^v^—Mn3—Al4^vi^	110.99 (5)	Al10^xxix^—Al7A—Al10^xxvi^	108.96 (8)
Al12—Mn3—Al4^vi^	110.99 (5)	Ni1^xxxvii^—Al7B—Ni1^xxxiii^	180.00 (4)
Al6—Mn3—Al4^vi^	108.13 (6)	Ni1^xxxvii^—Al7B—Al10^xxx^	57.62 (2)
Al3—Mn3—Al13^iv^	72.43 (4)	Ni1^xxxiii^—Al7B—Al10^xxx^	122.38 (2)
Al12^v^—Mn3—Al13^iv^	65.12 (4)	Ni1^xxxvii^—Al7B—Al10^xxxi^	122.38 (2)
Al12—Mn3—Al13^iv^	128.48 (5)	Ni1^xxxiii^—Al7B—Al10^xxxi^	57.62 (2)
Al6—Mn3—Al13^iv^	69.36 (3)	Al10^xxx^—Al7B—Al10^xxxi^	180.00 (6)
Al4^vi^—Mn3—Al13^iv^	106.82 (4)	Ni1^xxxvii^—Al7B—Al10^xxxii^	57.62 (2)
Al3—Mn3—Al13^iii^	72.43 (4)	Ni1^xxxiii^—Al7B—Al10^xxxii^	122.38 (2)
Al12^v^—Mn3—Al13^iii^	128.48 (5)	Al10^xxx^—Al7B—Al10^xxxii^	113.96 (5)
Al12—Mn3—Al13^iii^	65.12 (4)	Al10^xxxi^—Al7B—Al10^xxxii^	66.04 (5)
Al6—Mn3—Al13^iii^	69.36 (3)	Ni1^xxxvii^—Al7B—Al10^x^	122.38 (2)
Al4^vi^—Mn3—Al13^iii^	106.82 (4)	Ni1^xxxiii^—Al7B—Al10^x^	57.62 (2)
Al13^iv^—Mn3—Al13^iii^	132.84 (7)	Al10^xxx^—Al7B—Al10^x^	66.04 (5)
Al3—Mn3—Al5^vi^	124.43 (8)	Al10^xxxi^—Al7B—Al10^x^	113.96 (5)
Al12^v^—Mn3—Al5^vi^	63.87 (4)	Al10^xxxii^—Al7B—Al10^x^	180.00 (6)
Al12—Mn3—Al5^vi^	63.87 (4)	Mn2^xii^—Al8—Mn1^x^	170.33 (12)
Al6—Mn3—Al5^vi^	166.34 (6)	Mn2^xii^—Al8—Al7A^xii^	60.24 (7)
Al4^vi^—Mn3—Al5^vi^	58.21 (5)	Mn1^x^—Al8—Al7A^xii^	124.28 (6)
Al13^iv^—Mn3—Al5^vi^	112.78 (3)	Mn2^xii^—Al8—Al7A^xxxviii^	60.24 (7)
Al13^iii^—Mn3—Al5^vi^	112.78 (3)	Mn1^x^—Al8—Al7A^xxxviii^	124.28 (6)
Al3—Mn3—Al11^xvii^	121.85 (3)	Al7A^xii^—Al8—Al7A^xxxviii^	96.93 (11)
Al12^v^—Mn3—Al11^xvii^	61.18 (4)	Mn2^xii^—Al8—Al6^xxxix^	114.85 (7)
Al12—Mn3—Al11^xvii^	116.13 (5)	Mn1^x^—Al8—Al6^xxxix^	58.18 (6)
Al6—Mn3—Al11^xvii^	115.10 (3)	Al7A^xii^—Al8—Al6^xxxix^	163.78 (10)
Al4^vi^—Mn3—Al11^xvii^	58.97 (3)	Al7A^xxxviii^—Al8—Al6^xxxix^	92.78 (5)
Al13^iv^—Mn3—Al11^xvii^	58.86 (4)	Mn2^xii^—Al8—Al6^xl^	114.85 (7)
Al13^iii^—Mn3—Al11^xvii^	165.67 (5)	Mn1^x^—Al8—Al6^xl^	58.18 (6)
Al5^vi^—Mn3—Al11^xvii^	59.51 (3)	Al7A^xii^—Al8—Al6^xl^	92.78 (5)
Al3—Mn3—Al11^xviii^	121.85 (3)	Al7A^xxxviii^—Al8—Al6^xl^	163.78 (10)
Al12^v^—Mn3—Al11^xviii^	116.14 (5)	Al6^xxxix^—Al8—Al6^xl^	75.03 (9)
Al12—Mn3—Al11^xviii^	61.18 (4)	Mn2^xii^—Al8—Al9^xxxiii^	125.60 (11)
Al6—Mn3—Al11^xviii^	115.10 (3)	Mn1^x^—Al8—Al9^xxxiii^	64.07 (6)
Al4^vi^—Mn3—Al11^xviii^	58.97 (3)	Al7A^xii^—Al8—Al9^xxxiii^	86.07 (8)
Al13^iv^—Mn3—Al11^xviii^	165.67 (5)	Al7A^xxxviii^—Al8—Al9^xxxiii^	86.07 (8)
Al13^iii^—Mn3—Al11^xviii^	58.86 (4)	Al6^xxxix^—Al8—Al9^xxxiii^	107.60 (7)
Al5^vi^—Mn3—Al11^xviii^	59.51 (3)	Al6^xl^—Al8—Al9^xxxiii^	107.60 (7)
Al11^xvii^—Mn3—Al11^xviii^	108.34 (5)	Mn2^xii^—Al8—Al13^xxxiv^	52.45 (5)
Al3—Mn3—Al10^viii^	119.02 (6)	Mn1^x^—Al8—Al13^xxxiv^	120.86 (8)
Al12^v^—Mn3—Al10^viii^	114.67 (4)	Al7A^xii^—Al8—Al13^xxxiv^	111.92 (9)
Al12—Mn3—Al10^viii^	169.66 (5)	Al7A^xxxviii^—Al8—Al13^xxxiv^	58.77 (6)
Al6—Mn3—Al10^viii^	59.98 (4)	Al6^xxxix^—Al8—Al13^xxxiv^	62.71 (5)
Al4^vi^—Mn3—Al10^viii^	58.82 (4)	Al6^xl^—Al8—Al13^xxxiv^	105.48 (9)
Al13^iv^—Mn3—Al10^viii^	60.10 (4)	Al9^xxxiii^—Al8—Al13^xxxiv^	141.28 (5)
Al13^iii^—Mn3—Al10^viii^	114.44 (5)	Mn2^xii^—Al8—Al13^xxvii^	52.45 (5)
Al5^vi^—Mn3—Al10^viii^	108.59 (4)	Mn1^x^—Al8—Al13^xxvii^	120.86 (8)
Al11^xvii^—Mn3—Al10^viii^	61.50 (3)	Al7A^xii^—Al8—Al13^xxvii^	58.77 (6)
Al11^xviii^—Mn3—Al10^viii^	109.26 (4)	Al7A^xxxviii^—Al8—Al13^xxvii^	111.92 (9)
Al3—Mn3—Al10^vi^	119.02 (6)	Al6^xxxix^—Al8—Al13^xxvii^	105.48 (9)
Al12^v^—Mn3—Al10^vi^	169.66 (5)	Al6^xl^—Al8—Al13^xxvii^	62.71 (5)
Al12—Mn3—Al10^vi^	114.67 (4)	Al9^xxxiii^—Al8—Al13^xxvii^	141.28 (5)
Al6—Mn3—Al10^vi^	59.98 (4)	Al13^xxxiv^—Al8—Al13^xxvii^	73.12 (7)
Al4^vi^—Mn3—Al10^vi^	58.82 (4)	Mn2^xii^—Al8—Al10^x^	122.79 (6)
Al13^iv^—Mn3—Al10^vi^	114.44 (5)	Mn1^x^—Al8—Al10^x^	60.78 (5)
Al13^iii^—Mn3—Al10^vi^	60.10 (4)	Al7A^xii^—Al8—Al10^x^	63.50 (5)
Al5^vi^—Mn3—Al10^vi^	108.59 (4)	Al7A^xxxviii^—Al8—Al10^x^	138.12 (11)
Al11^xvii^—Mn3—Al10^vi^	109.26 (4)	Al6^xxxix^—Al8—Al10^x^	116.10 (9)
Al11^xviii^—Mn3—Al10^vi^	61.50 (3)	Al6^xl^—Al8—Al10^x^	58.07 (5)
Al10^viii^—Mn3—Al10^vi^	59.69 (5)	Al9^xxxiii^—Al8—Al10^x^	57.55 (4)
Al2^iii^—Mn4—Al12^iii^	70.30 (6)	Al13^xxxiv^—Al8—Al10^x^	161.13 (8)
Al2^iii^—Mn4—Al12^xix^	70.30 (6)	Al13^xxvii^—Al8—Al10^x^	89.92 (4)
Al12^iii^—Mn4—Al12^xix^	77.24 (6)	Mn2^xii^—Al8—Al10^xxxv^	122.79 (6)
Al2^iii^—Mn4—Al4	119.16 (6)	Mn1^x^—Al8—Al10^xxxv^	60.78 (5)
Al12^iii^—Mn4—Al4	112.57 (5)	Al7A^xii^—Al8—Al10^xxxv^	138.12 (11)
Al12^xix^—Mn4—Al4	167.76 (5)	Al7A^xxxviii^—Al8—Al10^xxxv^	63.50 (5)
Al2^iii^—Mn4—Al4^xiii^	119.16 (6)	Al6^xxxix^—Al8—Al10^xxxv^	58.07 (5)
Al12^iii^—Mn4—Al4^xiii^	167.76 (5)	Al6^xl^—Al8—Al10^xxxv^	116.10 (9)
Al12^xix^—Mn4—Al4^xiii^	112.57 (5)	Al9^xxxiii^—Al8—Al10^xxxv^	57.55 (4)
Al4—Mn4—Al4^xiii^	56.78 (7)	Al13^xxxiv^—Al8—Al10^xxxv^	89.92 (4)
Al2^iii^—Mn4—Al11^xix^	67.93 (4)	Al13^xxvii^—Al8—Al10^xxxv^	161.13 (8)
Al12^iii^—Mn4—Al11^xix^	130.89 (5)	Al10^x^—Al8—Al10^xxxv^	105.39 (8)
Al12^xix^—Mn4—Al11^xix^	65.30 (4)	Ni1—Al9—Ni1^xx^	114.80 (7)
Al4—Mn4—Al11^xix^	109.69 (5)	Ni1—Al9—Al10^xxi^	144.85 (7)
Al4^xiii^—Mn4—Al11^xix^	61.32 (4)	Ni1^xx^—Al9—Al10^xxi^	54.20 (3)
Al2^iii^—Mn4—Al11^iii^	67.93 (4)	Ni1—Al9—Al10^iii^	54.20 (3)
Al12^iii^—Mn4—Al11^iii^	65.30 (4)	Ni1^xx^—Al9—Al10^iii^	144.85 (7)
Al12^xix^—Mn4—Al11^iii^	130.89 (5)	Al10^xxi^—Al9—Al10^iii^	112.70 (8)
Al4—Mn4—Al11^iii^	61.32 (4)	Ni1—Al9—Al9^vii^	58.77 (3)
Al4^xiii^—Mn4—Al11^iii^	109.69 (5)	Ni1^xx^—Al9—Al9^vii^	58.77 (3)
Al11^xix^—Mn4—Al11^iii^	118.44 (6)	Al10^xxi^—Al9—Al9^vii^	110.28 (4)
Al2^iii^—Mn4—Al1	125.73 (7)	Al10^iii^—Al9—Al9^vii^	110.28 (4)
Al12^iii^—Mn4—Al1	67.95 (4)	Ni1—Al9—Mn4^iii^	108.60 (4)
Al12^xix^—Mn4—Al1	67.95 (4)	Ni1^xx^—Al9—Mn4^iii^	108.60 (4)
Al4—Mn4—Al1	108.06 (4)	Al10^xxi^—Al9—Mn4^iii^	106.47 (5)
Al4^xiii^—Mn4—Al1	108.06 (4)	Al10^iii^—Al9—Mn4^iii^	106.47 (5)
Al11^xix^—Mn4—Al1	120.10 (3)	Al9^vii^—Al9—Mn4^iii^	110.52 (4)
Al11^iii^—Mn4—Al1	120.10 (3)	Ni1—Al9—Al11	53.63 (3)
Al2^iii^—Mn4—Al5	121.80 (3)	Ni1^xx^—Al9—Al11	141.43 (7)
Al12^iii^—Mn4—Al5	62.05 (4)	Al10^xxi^—Al9—Al11	157.30 (8)
Al12^xix^—Mn4—Al5	124.60 (5)	Al10^iii^—Al9—Al11	64.15 (3)
Al4—Mn4—Al5	58.77 (5)	Al9^vii^—Al9—Al11	91.08 (4)
Al4^xiii^—Mn4—Al5	105.73 (4)	Mn4^iii^—Al9—Al11	56.79 (4)
Al11^xix^—Mn4—Al5	167.00 (5)	Ni1—Al9—Al11^xx^	141.43 (7)
Al11^iii^—Mn4—Al5	63.11 (4)	Ni1^xx^—Al9—Al11^xx^	53.63 (3)
Al1—Mn4—Al5	62.93 (4)	Al10^xxi^—Al9—Al11^xx^	64.16 (3)
Al2^iii^—Mn4—Al5^xiii^	121.80 (3)	Al10^iii^—Al9—Al11^xx^	157.30 (8)
Al12^iii^—Mn4—Al5^xiii^	124.60 (5)	Al9^vii^—Al9—Al11^xx^	91.08 (4)
Al12^xix^—Mn4—Al5^xiii^	62.05 (4)	Mn4^iii^—Al9—Al11^xx^	56.79 (4)
Al4—Mn4—Al5^xiii^	105.73 (4)	Al11—Al9—Al11^xx^	109.37 (7)
Al4^xiii^—Mn4—Al5^xiii^	58.77 (5)	Ni1—Al9—Al4^xxi^	148.57 (6)
Al11^xix^—Mn4—Al5^xiii^	63.11 (4)	Ni1^xx^—Al9—Al4^xxi^	96.50 (4)
Al11^iii^—Mn4—Al5^xiii^	167.00 (5)	Al10^xxi^—Al9—Al4^xxi^	57.76 (5)
Al1—Mn4—Al5^xiii^	62.93 (4)	Al10^iii^—Al9—Al4^xxi^	101.40 (7)
Al5—Mn4—Al5^xiii^	112.26 (5)	Al9^vii^—Al9—Al4^xxi^	148.15 (4)
Al2^iii^—Mn4—Al9^iii^	65.12 (7)	Mn4^iii^—Al9—Al4^xxi^	55.13 (5)
Al12^iii^—Mn4—Al9^iii^	119.85 (5)	Al11—Al9—Al4^xxi^	99.98 (7)
Al12^xix^—Mn4—Al9^iii^	119.85 (5)	Al11^xx^—Al9—Al4^xxi^	57.08 (4)
Al4—Mn4—Al9^iii^	62.64 (4)	Ni1—Al9—Al4^iii^	96.50 (4)
Al4^xiii^—Mn4—Al9^iii^	62.64 (4)	Ni1^xx^—Al9—Al4^iii^	148.57 (6)
Al11^xix^—Mn4—Al9^iii^	61.75 (3)	Al10^xxi^—Al9—Al4^iii^	101.40 (7)
Al11^iii^—Mn4—Al9^iii^	61.75 (3)	Al10^iii^—Al9—Al4^iii^	57.76 (5)
Al1—Mn4—Al9^iii^	169.14 (6)	Al9^vii^—Al9—Al4^iii^	148.15 (4)
Al5—Mn4—Al9^iii^	112.73 (4)	Mn4^iii^—Al9—Al4^iii^	55.13 (5)
Al5^xiii^—Mn4—Al9^iii^	112.73 (4)	Al11—Al9—Al4^iii^	57.08 (4)
Al2^iii^—Mn4—Mn4^xiv^	175.64 (6)	Al11^xx^—Al9—Al4^iii^	99.98 (7)
Al12^iii^—Mn4—Mn4^xiv^	112.93 (3)	Al4^xxi^—Al9—Al4^iii^	52.11 (7)
Al12^xix^—Mn4—Mn4^xiv^	112.93 (3)	Ni1—Al9—Al2	82.51 (4)
Al4—Mn4—Mn4^xiv^	57.21 (3)	Ni1^xx^—Al9—Al2	82.51 (4)
Al4^xiii^—Mn4—Mn4^xiv^	57.21 (3)	Al10^xxi^—Al9—Al2	123.19 (4)
Al11^xix^—Mn4—Mn4^xiv^	110.38 (3)	Al10^iii^—Al9—Al2	123.19 (4)
Al11^iii^—Mn4—Mn4^xiv^	110.38 (3)	Al9^vii^—Al9—Al2	60.21 (4)
Al1—Mn4—Mn4^xiv^	58.63 (3)	Mn4^iii^—Al9—Al2	50.30 (4)
Al5—Mn4—Mn4^xiv^	59.23 (2)	Al11—Al9—Al2	60.52 (4)
Al5^xiii^—Mn4—Mn4^xiv^	59.23 (2)	Al11^xx^—Al9—Al2	60.52 (4)
Al9^iii^—Mn4—Mn4^xiv^	110.52 (4)	Al4^xxi^—Al9—Al2	99.67 (6)
Mn2—Al1—Mn4	148.62 (3)	Al4^iii^—Al9—Al2	99.67 (6)
Mn2—Al1—Mn4^xiv^	148.63 (3)	Ni1—Al9—Mn1^iii^	111.21 (4)
Mn4—Al1—Mn4^xiv^	62.75 (6)	Ni1^xx^—Al9—Mn1^iii^	111.21 (4)
Mn2—Al1—Al5	126.65 (5)	Al10^xxi^—Al9—Mn1^iii^	58.46 (4)
Mn4—Al1—Al5	59.36 (5)	Al10^iii^—Al9—Mn1^iii^	58.46 (4)
Mn4^xiv^—Al1—Al5	59.36 (5)	Al9^vii^—Al9—Mn1^iii^	147.96 (4)
Mn2—Al1—Al5^xiii^	126.65 (5)	Mn4^iii^—Al9—Mn1^iii^	101.52 (6)
Mn4—Al1—Al5^xiii^	59.36 (5)	Al11—Al9—Mn1^iii^	106.89 (5)
Mn4^xiv^—Al1—Al5^xiii^	59.36 (5)	Al11^xx^—Al9—Mn1^iii^	106.89 (5)
Al5—Al1—Al5^xiii^	106.69 (10)	Al4^xxi^—Al9—Mn1^iii^	55.61 (5)
Mn2—Al1—Al12^xv^	102.72 (5)	Al4^iii^—Al9—Mn1^iii^	55.61 (5)
Mn4—Al1—Al12^xv^	103.75 (7)	Al2—Al9—Mn1^iii^	151.82 (8)
Mn4^xiv^—Al1—Al12^xv^	52.13 (3)	Ni1^iii^—Al10—Al4	105.45 (5)
Al5—Al1—Al12^xv^	107.16 (6)	Ni1^iii^—Al10—Al6^xii^	139.58 (5)
Al5^xiii^—Al1—Al12^xv^	56.09 (3)	Al4—Al10—Al6^xii^	103.57 (5)
Mn2—Al1—Al12^iii^	102.72 (5)	Ni1^iii^—Al10—Al6^xxiii^	123.31 (5)
Mn4—Al1—Al12^iii^	52.13 (3)	Al4—Al10—Al6^xxiii^	98.33 (5)
Mn4^xiv^—Al1—Al12^iii^	103.75 (7)	Al6^xii^—Al10—Al6^xxiii^	78.80 (7)
Al5—Al1—Al12^iii^	56.09 (3)	Ni1^iii^—Al10—Mn1	122.22 (5)
Al5^xiii^—Al1—Al12^iii^	107.16 (6)	Al4—Al10—Mn1	58.18 (5)
Al12^xv^—Al1—Al12^iii^	154.55 (10)	Al6^xii^—Al10—Mn1	55.26 (5)
Mn2—Al1—Al12^iv^	102.72 (5)	Al6^xxiii^—Al10—Mn1	114.14 (5)
Mn4—Al1—Al12^iv^	103.75 (7)	Ni1^iii^—Al10—Al9^iii^	61.91 (5)
Mn4^xiv^—Al1—Al12^iv^	52.13 (3)	Al4—Al10—Al9^iii^	62.38 (5)
Al5—Al1—Al12^iv^	56.09 (3)	Al6^xii^—Al10—Al9^iii^	109.94 (6)
Al5^xiii^—Al1—Al12^iv^	107.16 (6)	Al6^xxiii^—Al10—Al9^iii^	159.88 (7)
Al12^xv^—Al1—Al12^iv^	64.22 (5)	Mn1—Al10—Al9^iii^	62.12 (4)
Al12^iii^—Al1—Al12^iv^	109.74 (6)	Ni1^iii^—Al10—Al13^xxxvi^	62.42 (4)
Mn2—Al1—Al12^xix^	102.72 (5)	Al4—Al10—Al13^xxxvi^	98.14 (6)
Mn4—Al1—Al12^xix^	52.13 (3)	Al6^xii^—Al10—Al13^xxxvi^	138.97 (6)
Mn4^xiv^—Al1—Al12^xix^	103.75 (7)	Al6^xxiii^—Al10—Al13^xxxvi^	63.76 (5)
Al5—Al1—Al12^xix^	107.16 (6)	Mn1—Al10—Al13^xxxvi^	156.23 (6)
Al5^xiii^—Al1—Al12^xix^	56.09 (3)	Al9^iii^—Al10—Al13^xxxvi^	110.94 (6)
Al12^xv^—Al1—Al12^xix^	109.74 (6)	Ni1^iii^—Al10—Al7B^xli^	49.75 (3)
Al12^iii^—Al1—Al12^xix^	64.22 (5)	Al4—Al10—Al7B^xli^	155.17 (5)
Al12^iv^—Al1—Al12^xix^	154.55 (10)	Al6^xii^—Al10—Al7B^xli^	97.94 (4)
Mn2—Al1—Al13	49.10 (4)	Al6^xxiii^—Al10—Al7B^xli^	97.86 (4)
Mn4—Al1—Al13	107.71 (3)	Mn1—Al10—Al7B^xli^	129.61 (5)
Mn4^xiv^—Al1—Al13	144.48 (4)	Al9^iii^—Al10—Al7B^xli^	98.73 (5)
Al5—Al1—Al13	85.93 (4)	Al13^xxxvi^—Al10—Al7B^xli^	72.59 (4)
Al5^xiii^—Al1—Al13	148.52 (3)	Ni1^iii^—Al10—Al8^x^	88.21 (6)
Al12^xv^—Al1—Al13	148.29 (8)	Al4—Al10—Al8^x^	103.25 (6)
Al12^iii^—Al1—Al13	55.78 (3)	Al6^xii^—Al10—Al8^x^	57.93 (6)
Al12^iv^—Al1—Al13	103.80 (4)	Al6^xxiii^—Al10—Al8^x^	134.91 (7)
Al12^xix^—Al1—Al13	92.86 (4)	Mn1—Al10—Al8^x^	51.89 (5)
Mn2—Al1—Al13^v^	49.10 (4)	Al9^iii^—Al10—Al8^x^	60.42 (5)
Mn4—Al1—Al13^v^	144.49 (4)	Al13^xxxvi^—Al10—Al8^x^	147.50 (8)
Mn4^xiv^—Al1—Al13^v^	107.71 (3)	Ni1^iii^—Al10—Al10^v^	161.38 (3)
Al5—Al1—Al13^v^	85.93 (4)	Al4—Al10—Al10^v^	57.62 (3)
Al5^xiii^—Al1—Al13^v^	148.52 (3)	Al6^xii^—Al10—Al10^v^	57.99 (3)
Al12^xv^—Al1—Al13^v^	92.86 (4)	Al6^xxiii^—Al10—Al10^v^	58.04 (3)
Al12^iii^—Al1—Al13^v^	103.80 (4)	Mn1—Al10—Al10^v^	58.05 (3)
Al12^iv^—Al1—Al13^v^	55.79 (3)	Al9^iii^—Al10—Al10^v^	110.28 (4)
Al12^xix^—Al1—Al13^v^	148.29 (8)	Al13^xxxvi^—Al10—Al10^v^	109.46 (4)
Al13—Al1—Al13^v^	58.63 (6)	Al8^x^—Al10—Al10^v^	102.64 (6)
Mn2—Al1—Al13^xiii^	49.10 (4)	Ni1^iii^—Al10—Al7A^xl^	53.82 (5)
Mn4—Al1—Al13^xiii^	107.71 (3)	Al4—Al10—Al7A^xl^	144.61 (7)
Mn4^xiv^—Al1—Al13^xiii^	144.49 (4)	Al6^xii^—Al10—Al7A^xl^	86.56 (5)
Al5—Al1—Al13^xiii^	148.52 (3)	Al6^xxiii^—Al10—Al7A^xl^	116.97 (7)
Al5^xiii^—Al1—Al13^xiii^	85.93 (4)	Mn1—Al10—Al7A^xl^	105.45 (6)
Al12^xv^—Al1—Al13^xiii^	103.80 (4)	Al9^iii^—Al10—Al7A^xl^	82.25 (7)
Al12^iii^—Al1—Al13^xiii^	92.86 (4)	Al13^xxxvi^—Al10—Al7A^xl^	95.49 (6)
Al12^iv^—Al1—Al13^xiii^	148.29 (8)	Al8^x^—Al10—Al7A^xl^	53.56 (7)
Al12^xix^—Al1—Al13^xiii^	55.79 (3)	Al10^v^—Al10—Al7A^xl^	144.48 (4)
Al13—Al1—Al13^xiii^	70.31 (7)	Ni1^iii^—Al10—Mn3^xxiii^	104.59 (4)
Al13^v^—Al1—Al13^xiii^	98.19 (9)	Al4—Al10—Mn3^xxiii^	54.15 (4)
Mn4^xxii^—Al2—Mn4^iii^	171.29 (12)	Al6^xii^—Al10—Mn3^xxiii^	115.15 (5)
Mn4^xxii^—Al2—Al12	117.15 (7)	Al6^xxiii^—Al10—Mn3^xxiii^	52.94 (4)
Mn4^iii^—Al2—Al12	56.15 (4)	Mn1—Al10—Mn3^xxiii^	104.50 (4)
Mn4^xxii^—Al2—Al12^v^	56.15 (4)	Al9^iii^—Al10—Mn3^xxiii^	107.56 (5)
Mn4^iii^—Al2—Al12^v^	117.15 (7)	Al13^xxxvi^—Al10—Mn3^xxiii^	54.12 (4)
Al12—Al2—Al12^v^	61.08 (6)	Al8^x^—Al10—Mn3^xxiii^	156.03 (6)
Mn4^xxii^—Al2—Al12^vii^	56.15 (4)	Al10^v^—Al10—Mn3^xxiii^	60.16 (2)
Mn4^iii^—Al2—Al12^vii^	117.15 (7)	Al7A^xl^—Al10—Mn3^xxiii^	149.60 (6)
Al12—Al2—Al12^vii^	97.05 (9)	Ni1—Al11—Mn4^iii^	120.04 (5)
Al12^v^—Al2—Al12^vii^	66.82 (7)	Ni1—Al11—Al4^iii^	105.21 (5)
Mn4^xxii^—Al2—Al12^xx^	117.15 (7)	Mn4^iii^—Al11—Al4^iii^	58.05 (5)
Mn4^iii^—Al2—Al12^xx^	56.15 (4)	Ni1—Al11—Al13^vi^	63.18 (4)
Al12—Al2—Al12^xx^	66.82 (7)	Mn4^iii^—Al11—Al13^vi^	157.51 (6)
Al12^v^—Al2—Al12^xx^	97.05 (9)	Al4^iii^—Al11—Al13^vi^	99.47 (6)
Al12^vii^—Al2—Al12^xx^	61.08 (6)	Ni1—Al11—Al12^xviii^	120.70 (5)
Mn4^xxii^—Al2—Al9^vii^	64.57 (5)	Mn4^iii^—Al11—Al12^xviii^	118.86 (5)
Mn4^iii^—Al2—Al9^vii^	124.14 (9)	Al4^iii^—Al11—Al12^xviii^	99.45 (6)
Al12—Al2—Al9^vii^	145.81 (4)	Al13^vi^—Al11—Al12^xviii^	60.03 (4)
Al12^v^—Al2—Al9^vii^	108.81 (4)	Ni1—Al11—Al12	140.58 (6)
Al12^vii^—Al2—Al9^vii^	108.81 (4)	Mn4^iii^—Al11—Al12	53.81 (4)
Al12^xx^—Al2—Al9^vii^	145.82 (4)	Al4^iii^—Al11—Al12	100.93 (6)
Mn4^xxii^—Al2—Al9	124.14 (9)	Al13^vi^—Al11—Al12	139.80 (6)
Mn4^iii^—Al2—Al9	64.57 (5)	Al12^xviii^—Al11—Al12	82.62 (5)
Al12—Al2—Al9	108.81 (4)	Ni1—Al11—Al9	60.86 (4)
Al12^v^—Al2—Al9	145.81 (4)	Mn4^iii^—Al11—Al9	61.46 (4)
Al12^vii^—Al2—Al9	145.81 (4)	Al4^iii^—Al11—Al9	61.51 (5)
Al12^xx^—Al2—Al9	108.81 (4)	Al13^vi^—Al11—Al9	110.64 (6)
Al9^vii^—Al2—Al9	59.57 (9)	Al12^xviii^—Al11—Al9	158.50 (6)
Mn4^xxii^—Al2—Al11^xx^	122.17 (3)	Al12—Al11—Al9	109.52 (5)
Mn4^iii^—Al2—Al11^xx^	60.75 (3)	Ni1—Al11—Al2	85.94 (6)
Al12—Al2—Al11^xx^	112.13 (4)	Mn4^iii^—Al11—Al2	51.31 (3)
Al12^v^—Al2—Al11^xx^	153.77 (8)	Al4^iii^—Al11—Al2	102.30 (5)
Al12^vii^—Al2—Al11^xx^	90.36 (4)	Al13^vi^—Al11—Al2	145.99 (6)
Al12^xx^—Al2—Al11^xx^	59.07 (3)	Al12^xviii^—Al11—Al2	139.28 (7)
Al9^vii^—Al2—Al11^xx^	90.20 (6)	Al12—Al11—Al2	59.75 (6)
Al9—Al2—Al11^xx^	59.65 (4)	Al9—Al11—Al2	59.83 (6)
Mn4^xxii^—Al2—Al11^v^	60.75 (3)	Ni1—Al11—Al5^iii^	160.26 (6)
Mn4^iii^—Al2—Al11^v^	122.17 (3)	Mn4^iii^—Al11—Al5^iii^	59.85 (4)
Al12—Al2—Al11^v^	90.36 (4)	Al4^iii^—Al11—Al5^iii^	56.74 (5)
Al12^v^—Al2—Al11^v^	59.07 (3)	Al13^vi^—Al11—Al5^iii^	108.98 (6)
Al12^vii^—Al2—Al11^v^	112.13 (4)	Al12^xviii^—Al11—Al5^iii^	60.86 (5)
Al12^xx^—Al2—Al11^v^	153.77 (8)	Al12—Al11—Al5^iii^	57.49 (4)
Al9^vii^—Al2—Al11^v^	59.65 (4)	Al9—Al11—Al5^iii^	109.91 (6)
Al9—Al2—Al11^v^	90.19 (6)	Al2—Al11—Al5^iii^	104.78 (6)
Al11^xx^—Al2—Al11^v^	146.39 (10)	Ni1—Al11—Mn3^xviii^	104.64 (4)
Mn4^xxii^—Al2—Al11^vii^	60.75 (3)	Mn4^iii^—Al11—Mn3^xviii^	105.35 (5)
Mn4^iii^—Al2—Al11^vii^	122.17 (3)	Al4^iii^—Al11—Mn3^xviii^	54.22 (4)
Al12—Al2—Al11^vii^	153.77 (8)	Al13^vi^—Al11—Mn3^xviii^	54.73 (4)
Al12^v^—Al2—Al11^vii^	112.13 (4)	Al12^xviii^—Al11—Mn3^xviii^	52.49 (4)
Al12^vii^—Al2—Al11^vii^	59.07 (3)	Al12—Al11—Mn3^xviii^	114.57 (5)
Al12^xx^—Al2—Al11^vii^	90.36 (4)	Al9—Al11—Mn3^xviii^	106.02 (5)
Al9^vii^—Al2—Al11^vii^	59.65 (4)	Al2—Al11—Mn3^xviii^	155.83 (5)
Al9—Al2—Al11^vii^	90.19 (6)	Al5^iii^—Al11—Mn3^xviii^	59.41 (4)
Al11^xx^—Al2—Al11^vii^	61.59 (5)	Ni1—Al11—Al11^v^	54.29 (3)
Al11^v^—Al2—Al11^vii^	107.97 (6)	Mn4^iii^—Al11—Al11^v^	110.38 (3)
Mn4^xxii^—Al2—Al11	122.17 (3)	Al4^iii^—Al11—Al11^v^	150.42 (4)
Mn4^iii^—Al2—Al11	60.75 (3)	Al13^vi^—Al11—Al11^v^	89.65 (4)
Al12—Al2—Al11	59.07 (3)	Al12^xviii^—Al11—Al11^v^	109.41 (4)
Al12^v^—Al2—Al11	90.36 (4)	Al12—Al11—Al11^v^	89.35 (4)
Al12^vii^—Al2—Al11	153.77 (8)	Al9—Al11—Al11^v^	88.92 (4)
Al12^xx^—Al2—Al11	112.13 (4)	Al2—Al11—Al11^v^	59.20 (3)
Al9^vii^—Al2—Al11	90.19 (6)	Al5^iii^—Al11—Al11^v^	145.45 (4)
Al9—Al2—Al11	59.65 (4)	Mn3^xviii^—Al11—Al11^v^	144.17 (3)
Al11^xx^—Al2—Al11	107.97 (6)	Ni1—Al11—Al10^iii^	53.44 (3)
Al11^v^—Al2—Al11	61.59 (5)	Mn4^iii^—Al11—Al10^iii^	104.42 (5)
Al11^vii^—Al2—Al11	146.39 (10)	Al4^iii^—Al11—Al10^iii^	56.65 (5)
Mn3—Al3—Mn3^xx^	174.83 (12)	Al13^vi^—Al11—Al10^iii^	58.05 (4)
Mn3—Al3—Al6^xx^	128.61 (10)	Al12^xviii^—Al11—Al10^iii^	105.30 (5)
Mn3^xx^—Al3—Al6^xx^	56.56 (5)	Al12—Al11—Al10^iii^	156.83 (6)
Mn3—Al3—Al6	56.56 (5)	Al9—Al11—Al10^iii^	56.86 (4)
Mn3^xx^—Al3—Al6	128.61 (10)	Al2—Al11—Al10^iii^	115.42 (6)
Al6^xx^—Al3—Al6	72.05 (9)	Al5^iii^—Al11—Al10^iii^	106.83 (5)
Mn3—Al3—Al12^xx^	120.36 (8)	Mn3^xviii^—Al11—Al10^iii^	59.38 (4)
Mn3^xx^—Al3—Al12^xx^	55.46 (4)	Al11^v^—Al11—Al10^iii^	107.73 (3)
Al6^xx^—Al3—Al12^xx^	103.70 (4)	Mn4^iii^—Al12—Mn3	161.15 (6)
Al6—Al3—Al12^xx^	150.24 (3)	Mn4^iii^—Al12—Al5^iii^	64.16 (5)
Mn3—Al3—Al12^vii^	120.36 (8)	Mn3—Al12—Al5^iii^	133.76 (6)
Mn3^xx^—Al3—Al12^vii^	55.46 (4)	Mn4^iii^—Al12—Al13^iii^	122.41 (6)
Al6^xx^—Al3—Al12^vii^	103.70 (4)	Mn3—Al12—Al13^iii^	58.70 (4)
Al6—Al3—Al12^vii^	150.24 (3)	Al5^iii^—Al12—Al13^iii^	93.09 (6)
Al12^xx^—Al3—Al12^vii^	59.39 (6)	Mn4^iii^—Al12—Al11^xviii^	131.84 (6)
Mn3—Al3—Al12^v^	55.46 (4)	Mn3—Al12—Al11^xviii^	66.33 (4)
Mn3^xx^—Al3—Al12^v^	120.35 (8)	Al5^iii^—Al12—Al11^xviii^	67.68 (5)
Al6^xx^—Al3—Al12^v^	150.24 (3)	Al13^iii^—Al12—Al11^xviii^	58.79 (4)
Al6—Al3—Al12^v^	103.70 (4)	Mn4^iii^—Al12—Al11	60.89 (4)
Al12^xx^—Al3—Al12^v^	93.84 (8)	Mn3—Al12—Al11	128.46 (6)
Al12^vii^—Al3—Al12^v^	64.92 (6)	Al5^iii^—Al12—Al11	62.40 (5)
Mn3—Al3—Al12	55.46 (4)	Al13^iii^—Al12—Al11	152.13 (6)
Mn3^xx^—Al3—Al12	120.35 (8)	Al11^xviii^—Al12—Al11	97.38 (5)
Al6^xx^—Al3—Al12	150.24 (3)	Mn4^iii^—Al12—Al2	53.54 (3)
Al6—Al3—Al12	103.70 (4)	Mn3—Al12—Al2	113.47 (5)
Al12^xx^—Al3—Al12	64.92 (6)	Al5^iii^—Al12—Al2	109.70 (6)
Al12^vii^—Al3—Al12	93.84 (8)	Al13^iii^—Al12—Al2	145.60 (6)
Al12^v^—Al3—Al12	59.38 (6)	Al11^xviii^—Al12—Al2	153.67 (6)
Mn3—Al3—Al13^iv^	55.87 (3)	Al11—Al12—Al2	61.18 (5)
Mn3^xx^—Al3—Al13^iv^	125.40 (3)	Mn4^iii^—Al12—Al5^vi^	125.99 (6)
Al6^xx^—Al3—Al13^iv^	99.90 (7)	Mn3—Al12—Al5^vi^	63.87 (4)
Al6—Al3—Al13^iv^	60.04 (4)	Al5^iii^—Al12—Al5^vi^	97.44 (4)
Al12^xx^—Al3—Al13^iv^	147.01 (8)	Al13^iii^—Al12—Al5^vi^	108.09 (6)
Al12^vii^—Al3—Al13^iv^	92.89 (4)	Al11^xviii^—Al12—Al5^vi^	60.97 (4)
Al12^v^—Al3—Al13^iv^	55.79 (3)	Al11—Al12—Al5^vi^	65.61 (5)
Al12—Al3—Al13^iv^	103.07 (5)	Al2—Al12—Al5^vi^	94.51 (5)
Mn3—Al3—Al13^xxi^	125.40 (3)	Mn4^iii^—Al12—Al12^v^	112.93 (3)
Mn3^xx^—Al3—Al13^xxi^	55.87 (3)	Mn3—Al12—Al12^v^	55.32 (3)
Al6^xx^—Al3—Al13^xxi^	60.04 (4)	Al5^iii^—Al12—Al12^v^	151.29 (4)
Al6—Al3—Al13^xxi^	99.90 (7)	Al13^iii^—Al12—Al12^v^	110.01 (4)
Al12^xx^—Al3—Al13^xxi^	55.79 (3)	Al11^xviii^—Al12—Al12^v^	109.41 (4)
Al12^vii^—Al3—Al13^xxi^	103.07 (5)	Al11—Al12—Al12^v^	90.65 (4)
Al12^v^—Al3—Al13^xxi^	147.01 (8)	Al2—Al12—Al12^v^	59.46 (3)
Al12—Al3—Al13^xxi^	92.89 (4)	Al5^vi^—Al12—Al12^v^	59.92 (3)
Al13^iv^—Al3—Al13^xxi^	156.64 (10)	Mn4^iii^—Al12—Al3	108.70 (5)
Mn3—Al3—Al13^xxii^	125.40 (3)	Mn3—Al12—Al3	53.43 (4)
Mn3^xx^—Al3—Al13^xxii^	55.87 (3)	Al5^iii^—Al12—Al3	148.34 (6)
Al6^xx^—Al3—Al13^xxii^	60.04 (4)	Al13^iii^—Al12—Al3	63.77 (5)
Al6—Al3—Al13^xxii^	99.90 (7)	Al11^xviii^—Al12—Al3	111.56 (5)
Al12^xx^—Al3—Al13^xxii^	103.07 (5)	Al11—Al12—Al3	143.97 (6)
Al12^vii^—Al3—Al13^xxii^	55.79 (3)	Al2—Al12—Al3	84.56 (6)
Al12^v^—Al3—Al13^xxii^	92.89 (4)	Al5^vi^—Al12—Al3	109.80 (5)
Al12—Al3—Al13^xxii^	147.01 (8)	Al12^v^—Al12—Al3	60.31 (3)
Al13^iv^—Al3—Al13^xxii^	69.61 (5)	Mn4^iii^—Al12—Al1^iii^	59.92 (5)
Al13^xxi^—Al3—Al13^xxii^	105.46 (6)	Mn3—Al12—Al1^iii^	119.49 (6)
Mn3—Al3—Al13^iii^	55.87 (3)	Al5^iii^—Al12—Al1^iii^	61.05 (5)
Mn3^xx^—Al3—Al13^iii^	125.40 (3)	Al13^iii^—Al12—Al1^iii^	62.78 (6)
Al6^xx^—Al3—Al13^iii^	99.90 (7)	Al11^xviii^—Al12—Al1^iii^	95.91 (5)
Al6—Al3—Al13^iii^	60.04 (4)	Al11—Al12—Al1^iii^	110.32 (6)
Al12^xx^—Al3—Al13^iii^	92.89 (4)	Al2—Al12—Al1^iii^	105.59 (5)
Al12^vii^—Al3—Al13^iii^	147.01 (8)	Al5^vi^—Al12—Al1^iii^	154.32 (5)
Al12^v^—Al3—Al13^iii^	103.07 (5)	Al12^v^—Al12—Al1^iii^	144.87 (3)
Al12—Al3—Al13^iii^	55.79 (3)	Al3—Al12—Al1^iii^	88.24 (5)
Al13^iv^—Al3—Al13^iii^	105.46 (6)	Mn2—Al13—Mn3^iii^	149.28 (6)
Al13^xxi^—Al3—Al13^iii^	69.61 (5)	Mn2—Al13—Al7A	59.00 (7)
Al13^xxii^—Al3—Al13^iii^	156.64 (10)	Mn3^iii^—Al13—Al7A	134.09 (6)
Al4^xiii^—Al4—Mn3^xxiii^	179.94 (4)	Mn2—Al13—Al11^xxiii^	140.75 (7)
Al4^xiii^—Al4—Mn4^xiv^	61.61 (4)	Mn3^iii^—Al13—Al11^xxiii^	66.42 (4)
Mn3^xxiii^—Al4—Mn4^xiv^	118.34 (5)	Al7A—Al13—Al11^xxiii^	113.16 (8)
Al4^xiii^—Al4—Mn4	61.61 (4)	Mn2—Al13—Ni1^xxiii^	102.62 (5)
Mn3^xxiii^—Al4—Mn4	118.34 (5)	Mn3^iii^—Al13—Ni1^xxiii^	107.22 (5)
Mn4^xiv^—Al4—Mn4	65.57 (5)	Al7A—Al13—Ni1^xxiii^	58.62 (6)
Al4^xiii^—Al4—Mn1	61.94 (4)	Al11^xxiii^—Al13—Ni1^xxiii^	54.73 (4)
Mn3^xxiii^—Al4—Mn1	118.11 (6)	Mn2—Al13—Al12^iii^	117.03 (7)
Mn4^xiv^—Al4—Mn1	112.85 (5)	Mn3^iii^—Al13—Al12^iii^	56.18 (4)
Mn4—Al4—Mn1	112.85 (5)	Al7A—Al13—Al12^iii^	167.20 (7)
Al4^xiii^—Al4—Al5	113.51 (5)	Al11^xxiii^—Al13—Al12^iii^	61.18 (4)
Mn3^xxiii^—Al4—Al5	66.43 (6)	Ni1^xxiii^—Al13—Al12^iii^	113.74 (5)
Mn4^xiv^—Al4—Al5	63.50 (5)	Mn2—Al13—Al10^xxxvi^	130.87 (7)
Mn4—Al4—Al5	63.50 (5)	Mn3^iii^—Al13—Al10^xxxvi^	65.78 (4)
Mn1—Al4—Al5	175.45 (8)	Al7A—Al13—Al10^xxxvi^	72.73 (6)
Al4^xiii^—Al4—Al10	113.02 (4)	Al11^xxiii^—Al13—Al10^xxxvi^	65.72 (4)
Mn3^xxiii^—Al4—Al10	67.03 (5)	Ni1^xxiii^—Al13—Al10^xxxvi^	53.97 (4)
Mn4^xiv^—Al4—Al10	174.21 (8)	Al12^iii^—Al13—Al10^xxxvi^	112.08 (6)
Mn4—Al4—Al10	114.51 (3)	Mn2—Al13—Al8^xvi^	52.37 (6)
Mn1—Al4—Al10	61.53 (4)	Mn3^iii^—Al13—Al8^xvi^	107.45 (7)
Al5—Al4—Al10	122.04 (7)	Al7A—Al13—Al8^xvi^	56.17 (7)
Al4^xiii^—Al4—Al10^v^	113.02 (4)	Al11^xxiii^—Al13—Al8^xvi^	159.89 (6)
Mn3^xxiii^—Al4—Al10^v^	67.03 (5)	Ni1^xxiii^—Al13—Al8^xvi^	113.35 (6)
Mn4^xiv^—Al4—Al10^v^	114.51 (3)	Al12^iii^—Al13—Al8^xvi^	132.88 (7)
Mn4—Al4—Al10^v^	174.21 (8)	Al10^xxxvi^—Al13—Al8^xvi^	94.20 (5)
Mn1—Al4—Al10^v^	61.53 (4)	Mn2—Al13—Al13^v^	51.85 (3)
Al5—Al4—Al10^v^	122.04 (7)	Mn3^iii^—Al13—Al13^v^	156.42 (3)
Al10—Al4—Al10^v^	64.76 (6)	Al7A—Al13—Al13^v^	57.51 (4)
Al4^xiii^—Al4—Al11^iii^	113.18 (4)	Al11^xxiii^—Al13—Al13^v^	90.35 (4)
Mn3^xxiii^—Al4—Al11^iii^	66.80 (4)	Ni1^xxiii^—Al13—Al13^v^	58.13 (3)
Mn4^xiv^—Al4—Al11^iii^	116.83 (6)	Al12^iii^—Al13—Al13^v^	110.01 (4)
Mn4—Al4—Al11^iii^	60.63 (3)	Al10^xxxvi^—Al13—Al13^v^	109.45 (4)
Mn1—Al4—Al11^iii^	116.63 (4)	Al8^xvi^—Al13—Al13^v^	95.79 (6)
Al5—Al4—Al11^iii^	64.51 (4)	Mn2—Al13—Al6^iii^	108.64 (6)
Al10—Al4—Al11^iii^	66.64 (4)	Mn3^iii^—Al13—Al6^iii^	54.29 (4)
Al10^v^—Al4—Al11^iii^	122.34 (7)	Al7A—Al13—Al6^iii^	87.37 (5)
Al4^xiii^—Al4—Al11^iv^	113.18 (4)	Al11^xxiii^—Al13—Al6^iii^	109.27 (6)
Mn3^xxiii^—Al4—Al11^iv^	66.80 (4)	Ni1^xxiii^—Al13—Al6^iii^	109.41 (5)
Mn4^xiv^—Al4—Al11^iv^	60.63 (3)	Al12^iii^—Al13—Al6^iii^	105.27 (6)
Mn4—Al4—Al11^iv^	116.83 (6)	Al10^xxxvi^—Al13—Al6^iii^	57.61 (5)
Mn1—Al4—Al11^iv^	116.63 (4)	Al8^xvi^—Al13—Al6^iii^	56.54 (6)
Al5—Al4—Al11^iv^	64.51 (4)	Al13^v^—Al13—Al6^iii^	144.62 (4)
Al10—Al4—Al11^iv^	122.34 (7)	Mn2—Al13—Al1	58.45 (6)
Al10^v^—Al4—Al11^iv^	66.64 (4)	Mn3^iii^—Al13—Al1	115.78 (6)
Al11^iii^—Al4—Al11^iv^	120.83 (8)	Al7A—Al13—Al1	109.92 (7)
Al4^xiii^—Al4—Al9^xv^	63.95 (3)	Al11^xxiii^—Al13—Al1	96.52 (5)
Mn3^xxiii^—Al4—Al9^xv^	116.07 (4)	Ni1^xxiii^—Al13—Al1	109.98 (4)
Mn4^xiv^—Al4—Al9^xv^	62.23 (4)	Al12^iii^—Al13—Al1	61.44 (5)
Mn4—Al4—Al9^xv^	117.00 (6)	Al10^xxxvi^—Al13—Al1	160.56 (6)
Mn1—Al4—Al9^xv^	62.31 (4)	Al8^xvi^—Al13—Al1	103.19 (6)
Al5—Al4—Al9^xv^	116.32 (5)	Al13^v^—Al13—Al1	60.68 (3)
Al10—Al4—Al9^xv^	114.06 (7)	Al6^iii^—Al13—Al1	140.42 (6)
Al10^v^—Al4—Al9^xv^	59.86 (4)	Mn2—Al13—Al3^iii^	97.93 (5)
Al11^iii^—Al4—Al9^xv^	177.12 (7)	Mn3^iii^—Al13—Al3^iii^	51.70 (3)
Al11^iv^—Al4—Al9^xv^	61.41 (4)	Al7A—Al13—Al3^iii^	130.66 (8)
Al4^xiii^—Al4—Al9^iii^	63.95 (3)	Al11^xxiii^—Al13—Al3^iii^	110.78 (6)
Mn3^xxiii^—Al4—Al9^iii^	116.07 (4)	Ni1^xxiii^—Al13—Al3^iii^	158.58 (5)
Mn4^xiv^—Al4—Al9^iii^	117.00 (6)	Al12^iii^—Al13—Al3^iii^	60.44 (6)
Mn4—Al4—Al9^iii^	62.23 (4)	Al10^xxxvi^—Al13—Al3^iii^	107.22 (5)
Mn1—Al4—Al9^iii^	62.31 (4)	Al8^xvi^—Al13—Al3^iii^	74.99 (7)
Al5—Al4—Al9^iii^	116.32 (5)	Al13^v^—Al13—Al3^iii^	142.73 (3)
Al10—Al4—Al9^iii^	59.86 (4)	Al6^iii^—Al13—Al3^iii^	57.38 (6)
Al10^v^—Al4—Al9^iii^	114.06 (7)	Al1—Al13—Al3^iii^	85.90 (5)
Al11^iii^—Al4—Al9^iii^	61.41 (4)		

Symmetry codes: (i) *x*, *y*+1, *z*; (ii) −*x*, −*y*+2, −*z*+1; (iii) −*x*+1/2, −*y*+3/2, −*z*+1; (iv) *x*−1/2, −*y*+3/2, −*z*+1; (v) −*x*, *y*, *z*; (vi) *x*, *y*, *z*−1; (vii) −*x*, *y*, −*z*+1/2; (viii) −*x*, *y*, *z*−1; (ix) *x*−1/2, −*y*+1/2, *z*+1/2; (x) −*x*+1/2, −*y*+1/2, −*z*+1; (xi) −*x*, −*y*+1, *z*+1/2; (xii) −*x*, −*y*+1, −*z*+1; (xiii) *x*, *y*, −*z*+3/2; (xiv) −*x*, *y*, −*z*+3/2; (xv) *x*−1/2, −*y*+3/2, *z*+1/2; (xvi) *x*, −*y*+1, *z*+1/2; (xvii) *x*−1/2, −*y*+3/2, −*z*; (xviii) −*x*+1/2, −*y*+3/2, −*z*; (xix) −*x*+1/2, −*y*+3/2, *z*+1/2; (xx) *x*, *y*, −*z*+1/2; (xxi) −*x*+1/2, −*y*+3/2, *z*−1/2; (xxii) *x*−1/2, −*y*+3/2, *z*−1/2; (xxiii) *x*, *y*, *z*+1; (xxiv) −*x*, *y*, *z*+1; (xxv) −*x*+1/2, *y*+1/2, −*z*+1/2; (xxvi) *x*−1/2, *y*+1/2, *z*; (xxvii) *x*, −*y*+1, −*z*+1; (xxviii) −*x*, −*y*+2, −*z*+2; (xxix) −*x*+1/2, *y*+1/2, *z*; (xxx) −*x*+1/2, *y*−1/2, *z*−1; (xxxi) *x*−1/2, −*y*+1/2, −*z*+1; (xxxii) *x*−1/2, *y*−1/2, *z*−1; (xxxiii) *x*, *y*−1, *z*; (xxxiv) *x*, −*y*+1, *z*−1/2; (xxxv) −*x*+1/2, −*y*+1/2, *z*−1/2; (xxxvi) −*x*+1/2, −*y*+3/2, −*z*+2; (xxxvii) −*x*, −*y*+1, −*z*; (xxxviii) −*x*, −*y*+1, *z*−1/2; (xxxix) *x*+1/2, *y*−1/2, −*z*+1/2; (xl) *x*+1/2, *y*−1/2, *z*; (xli) *x*+1/2, *y*+1/2, *z*+1.
