# Supplementary material for: The Al61.49Mn11.35Ni4 phase in the Al–Mn–Ni system
Source: IUCrdata. 2022 Jan 14;7(Pt 1):x220038. doi: 10.1107/S2414314622000384 (PMC9028548; doi:10.1107/S2414314622000384)
Supplement: Supplementary file 3 [file x-07-x220038-sup3.docx]

SUPPLEMENTARY MATERIALS:

**Crystal structure of the Al61.49Mn11.35Ni4 phase in the Al—Mn—Ni system**

**Qifa Hu, Bin Wen and Changzeng Fan***

State Key Laboratory of Metastable Materials Science and Technology, Yanshan University,

Qinhuangdao 066004, P.R. China

*Correspondence email: [chzfan@ysu.edu.cn](mailto:chzfan@ysu.edu.cn)

In order to guide the crystal structure refinement process, the chemical compositions were examined quantitatively by energy dispersive X-ray spectroscopy (EDX) analysis attached to a Hitachi S-3400N SEM. The examined points are designated in Fig. S1 and the corresponding results are listed in Tab. S1. The small deviation relative to the results of refinement (80.00:14.84:5.16) of chemical composition is probably caused by the tilt of the single crystal surface to the incident beam. In addition, the conductive adhesives and glues may also result in the detected impurity elements of carbon. For ease of viewing, the proportions of Al, Mn and Ni are calculated and shown in last column of the Tab.S1. In order to compare with the previous structure model (Robinson, 1954), the transformation matrix that transform the original setting to the current standard setting was listed and the results were presented in Tab.S2.


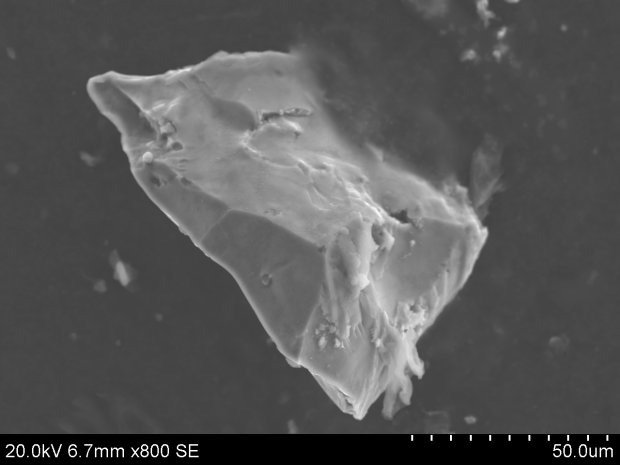


Fig. S1 single crystal of Al_61.49_Mn_11.35_Ni_4_

Tab. S1 EDS results for selected points as designated in Fig. S1

|  | Element | Weight(%) | Atomic(%) | Error(%) | Al:Mn:Ni |
| --- | --- | --- | --- | --- | --- |
| Spot 1 | C K | 26.58 | 47.08 | 11.09 | 84.04:11.60:4.37 |
|  | O K | 5.54 | 7.37 | 11.35 |  |
|  | AlK | 48.38 | 38.14 | 4.16 |  |
|  | MnK | 13.91 | 5.39 | 2.54 |  |
|  | NiK | 5.58 | 2.02 | 4.66 |  |

The transformation matrix:

$$\left( \begin{aligned} x' \\ y' \\ z' \end{aligned} \right)=\left( \begin{matrix} 0 & 0 & 1 \\ 1 & 0 & 0 \\ 0 & 1 & 0 \end{matrix} \right)\left( \begin{matrix} x \\ y \\ z \end{matrix} \right)$$

Where, (x, y, z) refers to the coordinates of the original setting and (xʹ, yʹ, zʹ) refers to the coordinates of the current setting.

Tab. S2 Comparison of atomic coordinates between the structure model of Al_60_Mn_11_Ni_4_(1954) [1] and the present structure model Al_61.49_Mn_11.35_Ni_4_ (2021)

| Original setting (1954) | | Current setting (1954) | | Current model (2021) | |
| --- | --- | --- | --- | --- | --- |
| Ni | 0.715 0.750 0.181 | Ni | 0.181 0.715 0.750 | Mn4 | 0.186 0.714 0.750 |
| Al1 | 0.553 0.935 0.188 | Al1 | 0.188 0.553 0.935 | Al10 | 0.190 0.554 0.934 |
| Al2 | 0.828 0.065 0.190 | Al2 | 0.190 0.828 0.065 | Al11 | 0.192 0.828 0.065 |
| Al3 | 0.714 0.127 0.190 | Al3 | 0.190 0.714 0.127 | Al12 | 0.187 0.716 0.126 |
| Al4 | 0.014 0.250 0.206 | Al4 | 0.206 0.014 0.250 | Al8 | 0.227 0.014 0.250 |
| Al5 | 0.891 0.885 0.185 | Al5 | 0.185 0.891 0.885 | Al13 | 0.189 0.891 0.886 |
| Al6 | 0.894 0.250 0.185 | Al6 | 0.185 0.894 0.250 | Al9 | 0.185 0.896 0.250 |
| Mn1 | 0.542 0.750 0.000 | Mn1 | 0.000 0.542 0.750 | Mn1 | 0.000 0.541 0.750 |
| Mn2 | 0.920 0.750 0.000 | Mn2 | 0.000 0.920 0.750 | Mn2 | 0.000 0.921 0.750 |
| Mn3 | 0.639 0.055 0.000 | Mn3 | 0.000 0.639 0.055 | Mn3 | 0.000 0.638 0.054 |
| Mn4 | 0.913 0.068 0.000 | Mn4 | 0.000 0.913 0.068 | Ni1 | 0.000 0.913 0.067 |
| Al7 | 0.637 0.853 0.000 | Al7 | 0.000 0.637 0.853 | Al4 | 0.000 0.638 0.849 |
| Al8 | 0.738 0.938 0.000 | Al8 | 0.000 0.738 0.938 | Al5 | 0.000 0.739 0.934 |
| Al9 | 0.813 0.750 0.000 | Al9 | 0.000 0.813 0.750 | Al1 | 0.000 0.811 0.750 |
| Al10 | 0.797 0.250 0.000 | Al10 | 0.000 0.797 0.250 | Al2 | 0.000 0.794 0.250 |
| Al11 | 0.538 0.122 0.000 | Al11 | 0.000 0.538 0.122 | Al6 | 0.000 0.539 0.117 |
| Al12 | 0.637 0.250 0.000 | Al12 | 0.000 0.637 0.250 | Al3 | 0.000 0.634 0.250 |
| Al13 | 0.988 0.897 0.000 | Al13 | 0.000 0.988 0.897 | Al7A | 0.000 0.985 0.906 |
|  |  |  |  | Al7B | 0.000 0.000 0.000 |

[1] Robinson, K. (1954). *Acta Cryst*. **7**, 494.
