# Supplementary material for: The Al61.49Mn11.35Ni4 phase in the Al–Mn–Ni system
Source: IUCrdata. 2022 Jan 14;7(Pt 1):x220038. doi: 10.1107/S2414314622000384 (PMC9028548; doi:10.1107/S2414314622000384)
Supplement: Supplementary file 4 [file x-07-x220038-sup4.docx]

i) General comment

The current study is a redetermination of a phase in the Al--Mn--Ni system. For better comparison it is advisable to refer to the original study by using the SAME setting of the space group and the same labelling and atomic coordinates. For your model you have used the standard setting of space group No 63 (Cmcm) whereas in the first study the unconventional setting Bbmm has been used (Robinson (1954), Acta Cryst. 7, 494)). It is tedious for the reader to compare two models with a different setting. Either you use the original setting (see attached PDF), or you should, at least, give the transformation matrix that transform the original setting to the current setting. Please revise or add details.

*In the revised manuscript, a note “(a detailed comparison of the coordinates between these two structure models along with the transformation matrix that transform the original setting to the current standard setting can be found in Tab.S2 of the supplementary materials)” was added in the second paragraph of the Structure description part*.

Please note also that the coordinates for the original study (Robinson, 1954) given in the supplementaries DO NOT refer to the originally published coordinates (see attached PDF). This should be corrected as well.

*Corrected.*

ii) Formula and formula units

The given formula with Z = 24 does not compare well with the original study. Please use Z = 2 and use the new formula throughout the manuscript and the supplementary material.

*Corrected.*

iii) Structure description

You wrote "Meanwhile, the polyhedron centred at Al3 atoms is not icosahedron but a pentaprism capped by two pentapyramids, as shown in Fig.3. The environments of Al3 can be found in Fig. 3 (b), where ten Al atoms (Al6, Al12 and Al13) and two Mn atoms (Mn3) are surrounding Al3 atom." This description is incomprehensible. A face is usually capped only by atom(s) not by other polyhedra. Please make this part more comprehensible.

*The phrase has been changed to“Meanwhile, the polyhedron centered at Al3 atoms is composed by a pentaprism and two pentapyramids, as shown in Fig.3. …”*

iv) References

Reference "Balanetskyy, S., Meisterernst, G., Grushko, B. & Feuerbacher, M. (2011). J. Alloys Compd. 509, 3795--3805." is listed in the reference list but is not cited, Please add citation or remove from the list.

*The reference is not closely related to the present work and has been deleted.*

v) Figures

In Figs. 2 and 3 the atom labels obstruct the atoms. Please replace the labels and their symmetry operators so that they do not mask atoms.

*Fig.2 and Fig3 have been modified.*
